# Differential Transcription Profiling Reveals the MicroRNAs Involved in Alleviating Damage to Photosynthesis under Drought Stress during the Grain Filling Stage in Wheat

**DOI:** 10.3390/ijms25105518

**Published:** 2024-05-18

**Authors:** Ruixiang Zhou, Yuhang Song, Xinyu Xue, Ruili Xue, Haifang Jiang, Yi Zhou, Xueli Qi, Yuexia Wang

**Affiliations:** 1 College of Life Sciences, Henan Agricultural University, 218 Ping’an Avenue, Zhengzhou 450046, China; 2 Henan Academy of Crop Molecular Breeding, Zhengzhou 450002, China

**Keywords:** wheat, drought, jasmonic acid (JA), abscisic acid (ABA), photosynthetic characteristics, microRNA (miRNA)

## Abstract

To explore the possible novel microRNA (miRNA) regulatory pathways in Zhengmai 1860, a newly cultivated drought-tolerant wheat (*Triticum aestivum* L.) cultivar, miRNA transcriptome sequencing of the flag leaves of Zhengmai 1860, drought-sensitive variety Zhoumai 18, and drought-resistant variety Bainong 207 was performed during the grain filling stage. We also observed changes in the chloroplast ultrastructure, phytohormone levels, and antioxidant- and photosynthesis-related physiological indicators in three wheat varieties. The results showed that the flag leaves of the drought-tolerant variety Zhengmai 1860 had higher chlorophyll contents and net photosynthetic rates than those of Zhoumai 18 under drought stress during the grain filling stage; in addition, the chloroplast structure was more complete. However, there was no significant difference between Zhengmai 1860 and Bainong 207. MiRNA transcriptome analysis revealed that the differential expression of the miRNAs and mRNAs exhibited variable specificity. The KEGG pathway enrichment results indicated that most of the genes were enriched in the MAPK signaling pathway, plant hormone signal transduction, photosynthetic antennae protein, and amino acid and carbohydrate metabolism. In the drought-tolerant cultivar Zhengmai 1860, tae-miR408 was targeted to regulate the allene oxide synthase (AOS) gene, inhibit its expression, reduce the AOS content, and decrease the synthesis of jasmonic acid (JA) and abscisic acid (ABA). The results of this study suggest that Zhengmai 1860 could improve the photosynthetic performance of flag leaves by inhibiting the expression of genes involved in the JA pathway through miRNAs under drought conditions. Moreover, multiple miRNAs may target chlorophyll, antioxidant enzymes, phytohormone signal transduction, and other related pathways; thus, it is possible to provide a more theoretical basis for wheat molecular breeding.

## 1. Introduction

Wheat (*Triticum aestivum* L.) is one of the three major food crops in the world and is widely planted worldwide. At present, the global food demand is growing rapidly, and by 2050, the food demand will have increased by 60–70% [1]. With the change in global climate, wheat is prone to drought during the grain filling period, which seriously affects wheat yield [2]. To alleviate the turbulence of drought on wheat yield, breeders have cultivated various wheat varieties with high yield and tolerance [3]. They can respond to drought stress at physiological and molecular levels and reduce the damage and yield reduction of wheat caused by drought. Drought stress results in the production of a large number of reactive oxygen species, destroys the photosynthetic structure of wheat plants, accelerates the senescence of wheat plants at the filling stage, and shortens the filling time [4]. The photosynthetic capacity of wheat plants at the grain filling stage directly affects yield. Under drought conditions, a high net photosynthetic rate and complete chloroplast structure are also indicators of strong drought resistance and high yield [5].

Abscisic acid (ABA), jasmonic acid (JA), brassinosterol (BR), cytokinin (CTK), and other phytohormones affect plant drought tolerance by regulating the expression of related genes [6]. ABA and JA can induce the MAPK signaling pathway to adapt to drought [7]. When plants are deprived of water, ABA and JA can regulate stomatal closure to reduce water loss [7]. JA can regulate the level of ABA by controlling the expression level of the genes involved in ABA synthesis and decomposition [8]. However, JA and ABA, as senescence-promoting phytohormones, can promote leaf senescence by inducing the expression of senescence-related genes (SAGs) or chlorophyll decomposition genes [9]. A study of BR revealed that it is also a pro-aging phytohormone that cooperates with ABA in response to drought. When maize is subjected to drought, BRs cause the accumulation of osmotic substances [10]. However, excessive JA and ABA cause premature leaf senescence, thus affecting the photosynthesis of wheat and leading to a decrease in yield [11]. In arid environments, CTK can delay leaf senescence through isoamyl transferase activity and negatively regulate the effects of ABA and JA to maintain normal plant growth and development. The expression of allene oxide synthase (AOS), a key enzyme in the JA synthesis pathway [8], is induced in wheat plants under abiotic stress, promoting the synthesis of JA [12]. However, its regulatory mechanism in the wheat drought response during grain filling has not been well elucidated.

MicroRNAs (miRNAs) are involved in a variety of plant regulatory pathways to cope with abiotic stress [13]. Studies have shown that the differential expression of miR408 can regulate the expression of anthocyanin-related genes and affect crop yield under drought stress [14]. In the JA biosynthesis pathway, a novel m0915-3p [15] targets the expression of the AOC gene, a key enzyme in JA biosynthesis in rice, but the miRNA that regulates AOS in wheat has not been found [16]. miRNAs are highly conserved, and their expression is related to tissue specificity and timing. The expressions of miRNAs in different varieties of drought-resistant wheat are normally not identical, and the expressions of miRNAs in different tissues at different stages are also very different. Therefore, identifying the miRNAs expressed in different resistant wheat varieties using transcriptome sequencing technology and exploring their roles in the regulation of target genes and physiological processes, such as senescence under drought stress, are highly important.

In this study, a newly bred drought-tolerant wheat variety, Zhengmai 1860, was used in combination with two other widely planted cultivars to compare its performance in response to drought stress at the grain filling stage. Transcriptome sequencing technology was used to screen the differentially expressed genes (DE genes) and miRNAs (DE miRNAs) under drought conditions. Functional enrichment was carried out to explore the possible miRNAs involved in the drought-related pathways; the aim was to identify a novel miRNA-mRNA regulatory pathway that might participate in drought resistance in this new wheat variety.

## 2. Results

### 2.1. Effects of Drought Stress at the Grain Filling Stage on the Chlorophyll Content and Photosynthetic Parameters of Different Wheat Varieties

**Figure 1:**
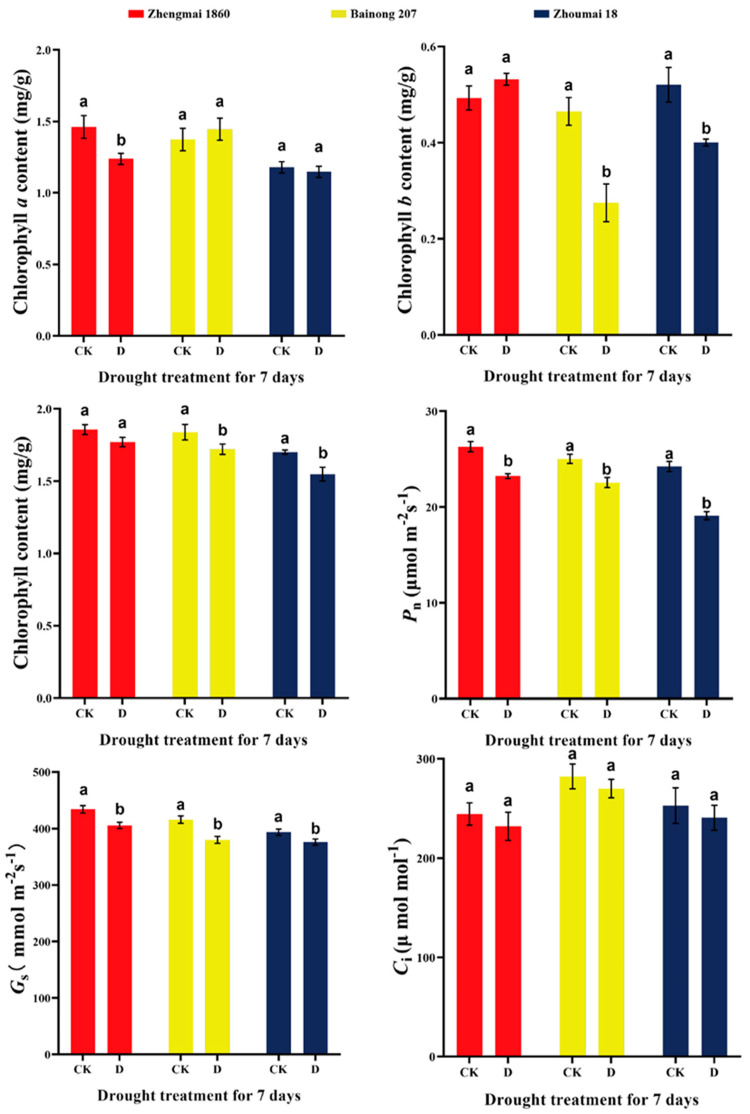
Chlorophyll content and net photosynthetic rate of flag leaves of different wheat varieties under drought stress at the grain filling stage. CK: control group; D: treatment group (drought for 7 days); *P*_n_: net photosynthetic rate; *C*_i_: intercellular CO_2_ concentration; *g*_s_: Stomatal conductance. The data in the figure are presented as the average value with standard deviation (SD) (n = 3). Data were analyzed using the one-way repeated measures analysis of variance (ANOVA) multiple comparison test and Sidak multiple comparison post hoc test (*p* < 0.05). The use of the same letter indicates no significant difference between samples, while the presence of different letters signifies significant differences.

Figure 1 shows that under drought stress during the grain filling stage, the chlorophyll *a* content decreased markedly in Zhengmai 1860, while the chlorophyll *b* content decreased significantly in Bainong 207 and Zhoumai 18 (*p* < 0.05). Similarly, compared with that in the control, the total chlorophyll contents in the leaves of Bainong 207 and Zhoumai 18 were reduced by 6.4% and 8.9%, respectively (*p* < 0.05). Moreover, compared with that in the control, the total chlorophyll content in Zhengmai 1860 was not obviously lower. The photosynthetic rate (*P*_n_) and stomatal conductance (*g*_s_) values decreased by 21% and 8.5% (Figure 1), respectively, in the Zhoumai 18 cultivar, while comparatively lower, though significant, decreases were detected in the Zhengmai 1860 and Bainong 207 cultivars (*p* < 0.05). However, there was only a slight, but not obvious, decrease in the CO_2_ concentration (*C*_i_) value under drought treatment. Taken together, the results indicated that the photosynthesis of Zhengmai 1860 and Bainong 207, when compared with that of Zhoumai 18, was better maintained under drought stress in the early and middle stages of grain filling.

### 2.2. Effects of Drought Stress during Grain Filling on the MDA Content and Antioxidant Enzyme Activities in the Leaves of Different Wheat Varieties

**Figure 2:**
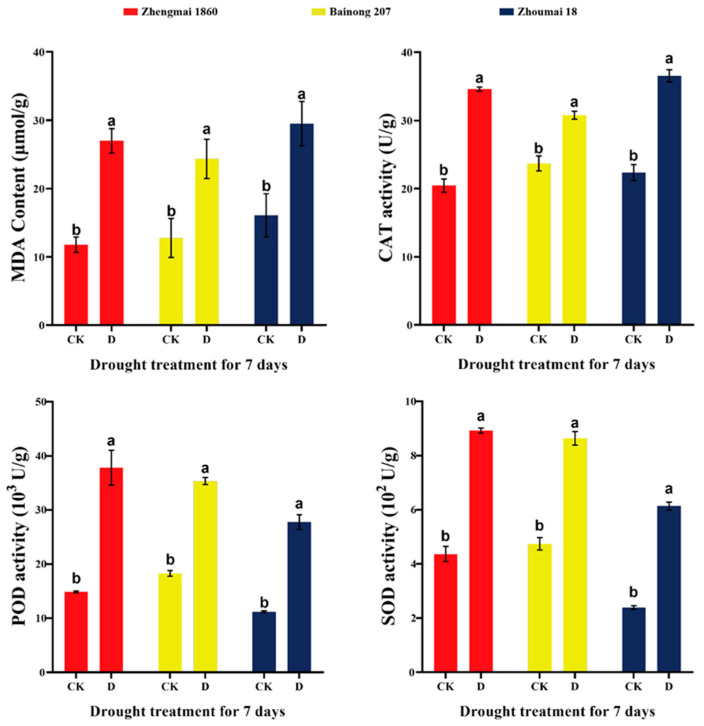
Catalase (CAT), Peroxidase (POD), and Superoxide Dismutase (SOD) activities and Malondialdehyde (MDA) content of different wheat varieties in the control group and drought group. CK: control group; D: treatment group (drought for 7 days). The data in the figure are presented as the average value with standard deviation (SD) (n = 3). Data were analyzed using the one-way repeated measures ANOVA multiple comparison test and Sidak multiple comparison post hoc test (*p* < 0.05). The use of the same letter indicates no significant difference between samples, while the presence of different letters signifies significant differences.

Under drought stress, a significant increase in Malondialdehyde (MDA) content was found in the flag leaves of the three wheat varieties at the filling stage (Figure 2), indicating that the three wheat varieties were subjected to oxidative damage under drought stress. However, the Catalase (CAT) and Peroxidase (POD) activities of Zhengmai 1860, which were the highest among the three cultivars, improved by 69% and 155%, respectively (*p* < 0.05), in response to drought stress (Figure 2). Similarly, the Superoxide Dismutase (SOD) activities of Zhengmai 1860, Bainong 207, and Zhoumai 18 increased by 1.05, 0.82, and 1.57 times, respectively. The results indicated that Zhengmai 1860 could reduce oxidative damage by increasing the activity of antioxidant enzymes under drought stress [17].

### 2.3. Effect of Drought on the Chloroplast Ultrastructure of Three Wheat Varieties

**Figure 3:**
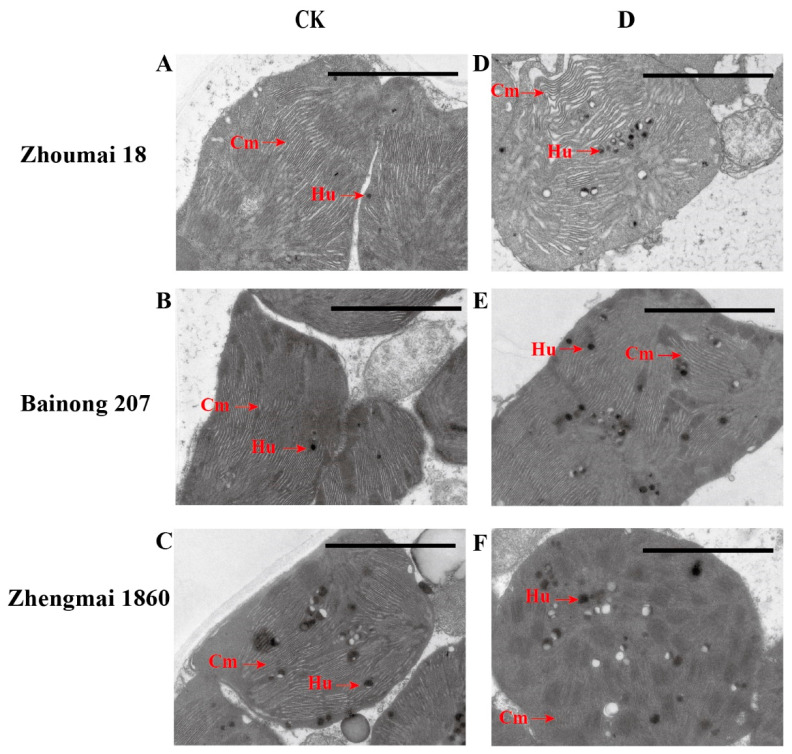
Ultrastructure of chloroplasts on the flag leaves of different wheat varieties at the grain filling stage. (**A**–**C**) refer to Zhoumai 18, Bainong 207, and Zhengmai 1860, respectively, in the CK group; (**D**–**F**) represent Zhoumai 18, Bainong 207, and Zhengmai 1860, respectively, in the D group. Cm: thylakoid; Hu: osmiophilic granule; scale: 2 μm.

Chloroplasts are essential for plant photosynthesis, and their structural stability has a great impact on photosynthesis [18]. TEM revealed that the thylakoid lamellae of Zhoumai 18 plants in the drought group were loosely arranged compared with those in the control group, and a large number of osmiophilic particles appeared (Figure 3). Comparatively, the thylakoid layers of the Bainong 207 plants were tightly arranged with more osmiophilic particles than those of the CK plants. In Zhengmai 1860, the thylakoid lamellae were still arranged clearly and in an orderly way and were stacked closely, and the number of osmiophilic particles did not increase markedly compared with that in the CK. The results showed that Zhengmai 1860 could maintain an intact chloroplast structure for a longer time under drought stress, compared with those of the other two varieties, thereby providing a better place for late photosynthesis [19].

### 2.4. Identification of Known microRNAs (miRNAs) and New miRNAs in Different Wheat Varieties

In this study, we analyzed the mRNA and miRNA transcriptomes of three wheat cultivars in response to drought stress. To identify the known miRNAs and new miRNAs in wheat, the sequences of miRNA precursors and mature miRNAs in wheat from the miRBase database were compared with the reads in the reference genome, and the new miRNA sequences were predicted using miReap version 0.2. A total of 401 known miRNAs and 109 known (conserved) miRNAs belonging to 35 miRNA families were obtained; *miR-1122* was the largest family, with 7 miRNA members. The second was miR-1120, which had six members, and miR-9657 and miR-966 both had four family members. According to the hairpin structure of the miRNA precursors, 292 new miRNAs were predicted (Supplementary Table S3).

### 2.5. Identification and Screening of Drought Stress-Related miRNAs and Genes in Different Wheat Varieties

**Figure 4:**
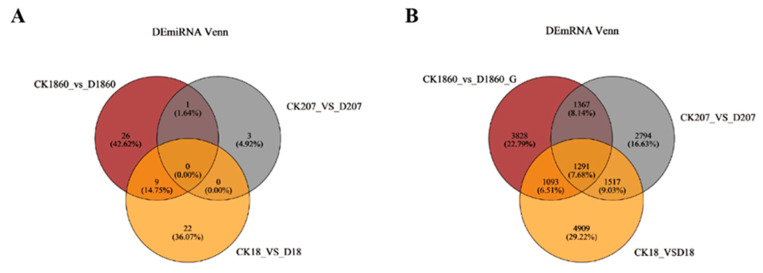
Comparison of drought stress-related microRNAs (miRNAs) and related genes in different wheat varieties using a Venn diagram. (**A**): Venn diagram analysis of drought related miRNAs; (**B**): Venn diagram analysis of drought related mRNAs. Circles of different colors represent genes in a group of gene sets, and the cross region of the circle represents the number of common genes among the gene sets.

Through differential expression analysis of DeSeq2 version 1.24.0, we screened the DE miRNAs and DE genes under drought stress. Venn diagram clustering analysis of the DE miRNAs and DE genes of the three varieties was also performed (Figure 4). A total of 83.63% of the DE miRNAs in each variety exhibited variety specificity, and the variety-specific DE genes were also more common than the common DE genes. Therefore, the differences in drought resistance among the three varieties were closely related to the specific DE genes.

### 2.6. Analysis of DE Gene Enrichment in Different Wheat Varieties under Drought Stress

In this study, plant-specific target gene prediction software (psRobot version v1.2) was used to predict the target genes of the miRNAs. Of a total of 401 miRNAs, 392 predicted 4172 target genes, 104 of the 109 known miRNAs predicted 4172 target genes, and 288 of the 292 new miRNAs predicted 3843 target genes.

#### 2.6.1. GO and KEGG Pathway Enrichment Analyses of Common Differentially Expressed Genes (DE Genes) under Drought Stress

**Figure 5:**
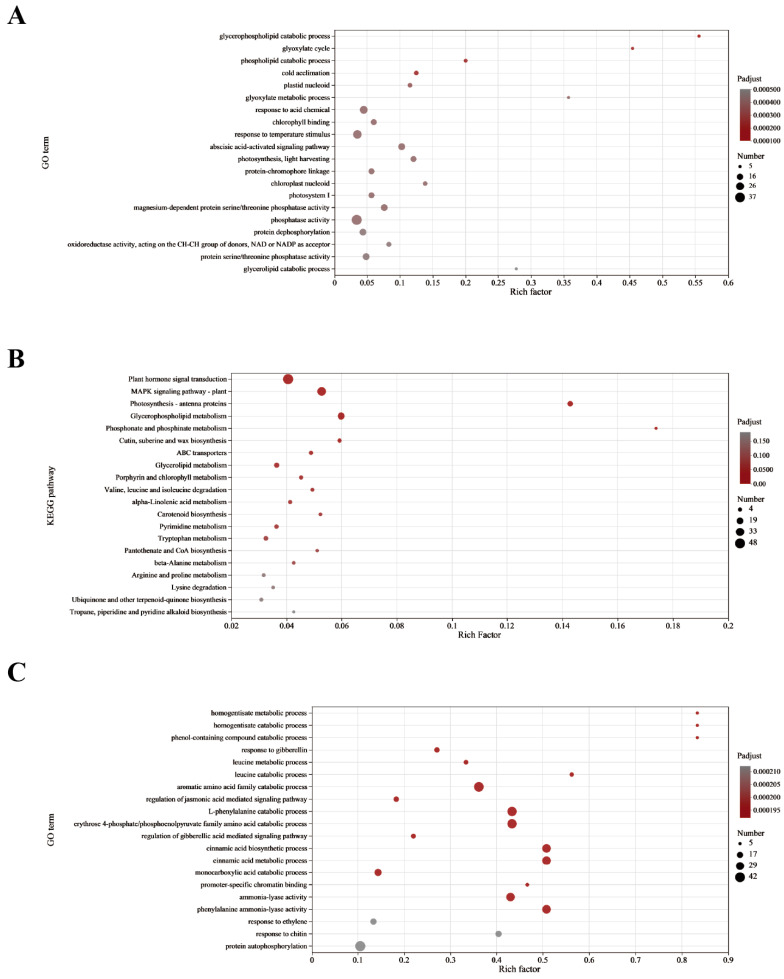

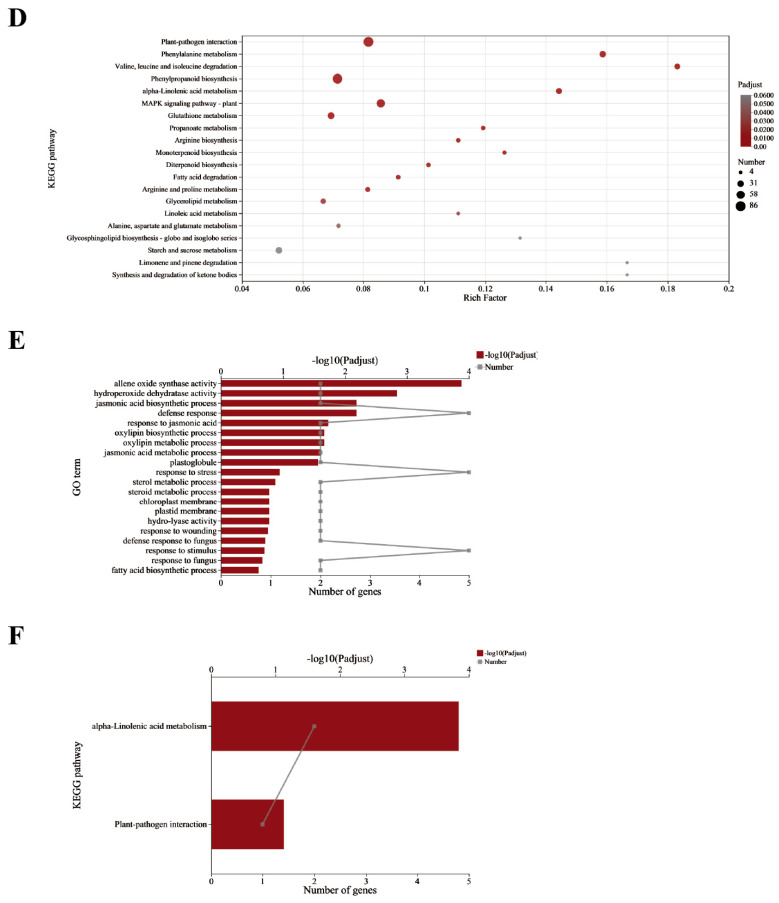
GO and KEGG enrichment analyses of the DE gene sets. (**A**,**B**) are the common differentially expressed genes (DE genes); (**C**,**D**) are the unique DE genes of Zhengmai 1860; (**E**,**F**) are the specific differentially expressed target genes of Zhengmai 1860. The vertical axis represents the name of the path; the horizontal axis represents the enrichment factor (the ratio of the number of genes enriched in the path to the number of annotated genes); the size of the point represents the number of genes in the path; and the color of the point corresponds to different *p* value ranges. The ordinate represents the GO term, and the lower abscissa represents the number of genes associated with the GO term in the comparison, corresponding to different points on the polyline. The upper abscissa represents the significance level of the enrichment, corresponding to the height of the column.

Statistics were generated for 20 GO function terms with high enrichment (Figure 5). The associated genes were enriched mainly in biological processes, such as phospholipid catabolism, the glyoxylic acid cycle, the response to temperature stimulation, the ABA-activated signaling pathway, and photosynthesis. According to the KEGG pathway enrichment analysis, the majority of the genes were enriched in plant hormone signal transduction, the MAPK signaling pathway, amino acid metabolism, carbohydrate metabolism, lipid metabolism, and the biosynthesis of secondary metabolite phenylpropanoids, indicating that these metabolic pathways generally play important roles in the wheat response to drought.

#### 2.6.2. Enrichment Analysis of Specific DE Genes and GO and KEGG Pathways of Drought-Tolerant Varieties

The results of the photosynthetic and oxidative index test revealed that Zhengmai 1860 had an obviously greater tolerance to drought than Bainong 207 and Zhoumai 18 [20]. Therefore, a GO functional enrichment analysis was carried out for the DE genes of Zhengmai 1860. The 20 GO function items with high enrichment were counted (Figure 5). The genes were enriched mainly in biological processes, such as urinary melanic acid metabolism, amino acid metabolism, and the regulation of JA-mediated signaling pathways. The DE genes were enriched in KEGG pathways, and 20 KEGG pathways with high enrichment were generated. The pathways involved were concentrated in specific pathways, such as catalase, RNA degradation, and nitrogen metabolism, and the common enrichment pathways, ABC transporters, the MAPK signaling pathways of plants, plant hormone signal transduction, and the amino acids, carbohydrates, lipids, other secondary metabolites, cofactors and vitamins, other amino acids, terpenes, and polyketides were also enriched in the metabolism. The Venn diagram analysis revealed that there was a greater species-specific expression of DE genes, and the metabolic pathways enriched by these specific DE genes may be an important reason for the obvious differences between Zhengmai 1860 and the other two varieties.

### 2.7. Correlation Analysis between Specific Differentially Expressed miRNAs (DE Genes) and Target Genes in Drought-Tolerant Varieties

A total of 26 miRNAs were differentially expressed under drought stress in Zhengmai 1860, and 546 target genes were predicted (Figure 5). A total of 773 miRNA-mRNA pairs were found with correlation analysis of the mRNA expression profiles, of which 122 pairs were markedly different (*p* < 0.05). The expression of all the target genes of the 122 pairs of miRNAs and mRNAs was compared with that of the differentially expressed genes of Zhengmai 1860 under drought stress. A total of eight target genes also exhibited significant changes in mRNA expression. After GO enrichment analysis of these eight genes, they were found to be enriched in biological processes, such as JA biosynthesis and metabolism-related pathways; stress response; fatty acid biosynthesis; cell components, such as plastid spheres, chloroplast membranes, and plastid membranes; and the activities of AOS, hydroperoxide dehydratase, and hydrolase. Moreover, according to the KEGG pathway analysis, two genes were enriched in the linolenic acid metabolism pathway, and the expression of tae-miR408, which targets this gene, was negatively correlated with the expression of these genes.

### 2.8. qRT-PCR Validation of mRNA and miRNA Expression Profile Data

**Figure 6:**
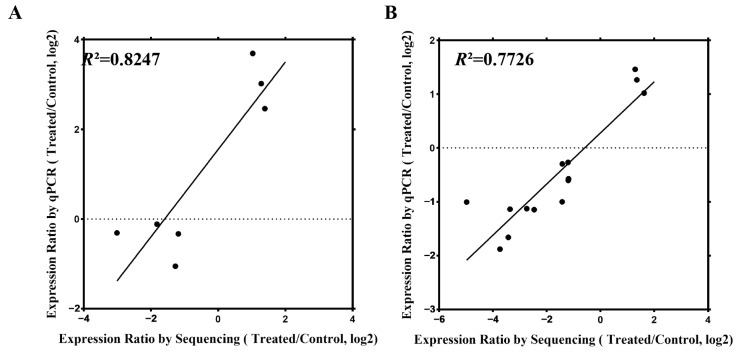
qRT-PCR validation of the transcriptome data. (**A**), linear regression analysis between miRNA expression and transcriptome data; (**B**), linear regression analysis between mRNA expression and transcriptome data; *R*^2^ is the correlation coefficient (goodness of fit).

To verify the reliability of the RNA-seq data, 18 target genes that were randomly selected from the miRNA-mRNA pairs with obvious regulatory relationships were analyzed using qRT-PCR; these genes included 8 target genes in the miRNA-mRNA pairs with specific differential expression of Zhengmai 1860 and 10 other target genes with *R*^2^ = 0.7726. Similarly, five randomly selected miRNAs and five miRNAs corresponding to the specific effect of Zhengmai 1860 were subjected to qRT-PCR, with *R*^2^ = 0.8247, which proved that the two had a high level of consistency (Figure 6).

### 2.9. Effects of Drought Stress on the Contents of Brassinosterol (BR), Cytokinin (CTK), Jasmonic Acid (JA), and Abscisic Acid (ABA) in Different Wheat Varieties

**Figure 7:**
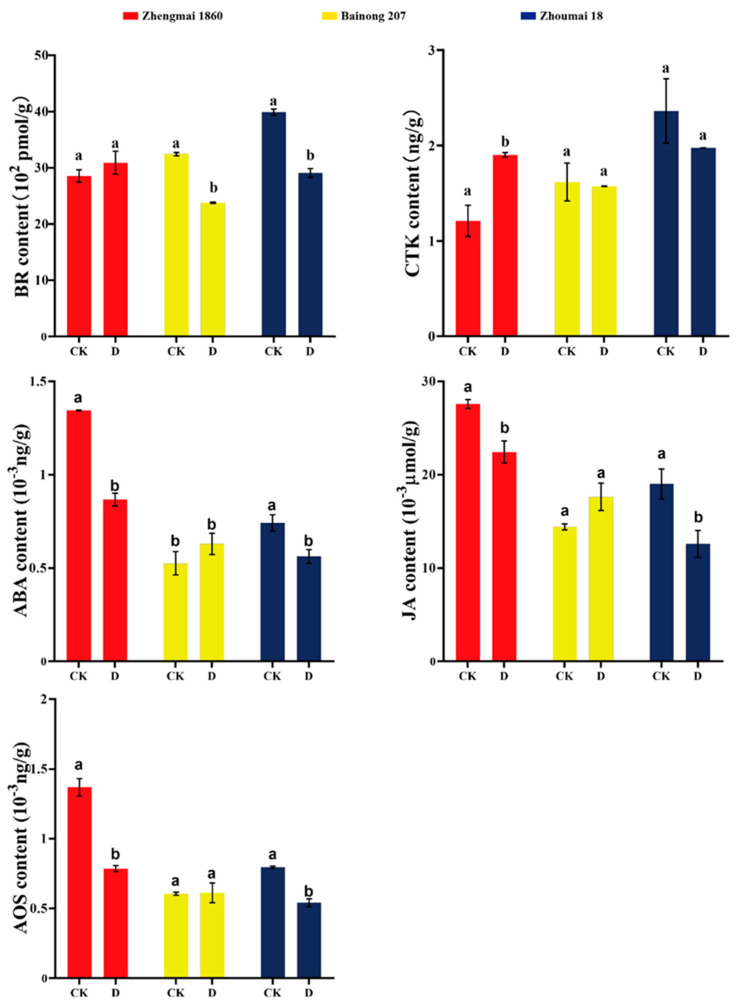
The brassinosterol (BR), cytokinin (CTK), abscisic acid (ABA), jasmonic acid (JA), and allene oxide synthase (AOS) contents of different wheat varieties under drought stress at the grain filling stage. CK: control group; D: treatment group (drought for 7 days). The data in the figure are presented as the average value with standard deviation (SD) (n = 3). Data were analyzed using the one-way repeated measures ANOVA multiple comparison test and Sidak multiple comparison post hoc test (*p* < 0.05). The use of the same letter indicates no significant difference between samples, while the presence of different letters signifies significant differences.

Under drought stress, the BR content in Zhengmai 1860 increased slightly but not obviously, while that in Bainong 207 and Zhoumai 18 significantly decreased (*p* < 0.05) (Figure 7). The CTK content in Zhengmai 1860 obviously increased in response to drought stress, but there was no marked change in the other two cultivars. The AOS, JA, and ABA contents in Zhengmai 1860 and Zhoumai 18 all decreased significantly under drought stress (*p* < 0.05), but the changes in Bainong 207 were not significant.

### 2.10. TaAOS Is the Target Gene of tae-miR408

**Figure 8:**
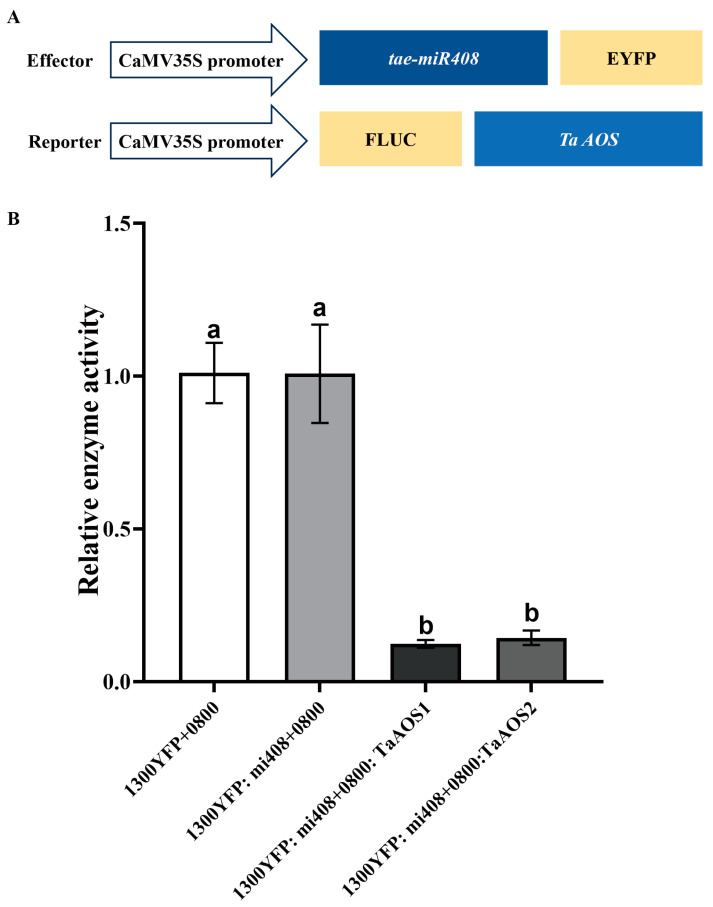
Verification of the targeted binding of tae-miR408 to *TaAOS* with dual fluorescein enzyme assay. (**A**), Schematic diagram of the effector and reporter. (**B**), Relative enzyme activity. The data in the figure are the average values (n = 3). Data were analyzed using the one-way repeated measures ANOVA multiple comparison test and Sidak multiple comparison post hoc test (*p* < 0.05). The use of the same letter indicates no significant difference between samples, while the presence of different letters signifies significant differences.

Through psRobot prediction, we found that tae-miR408 could bind to the *TaAOS* sequence. Thereafter, the interaction between tae-miR408 and *TaAOS* was further confirmed by dual-luciferase analysis (Figure 8). As shown, the fluorescence ratio of the control group (1300YFP + 0800) and the target gene (1300YFP: *miR408* + 0800) control group did not change, while the fluorescence value of the 1300YFP: *miR408* + 0800: *TaAOS* group decreased. However, the AOS content in Zhengmai 1860 in the drought group was lower than that in the control group (Figure 7). These results confirmed that the tae-miR408-targeted *TaAOS* sequence negatively regulated its expression under drought, resulting in a decrease in AOS content.

## 3. Discussion

### 3.1. miRNA-Triggered Chlorophyll Degradation Affects Photosynthetic Capacity under Drought Stress

Chlorophyll is an important component of the thylakoid membrane [21], and the total chlorophyll content is closely related to light absorption capacity [22]. Drought causes premature senescence of leaves at the grain filling stage, during which chlorophyll is degraded [23]. Under drought conditions, ABA plays an important role in mediating chlorophyll metabolism by regulating *MAPKKK18* [24]. It has been reported that wheat plants suffer drought during the grain filling period due to chlorophyll degradation, and this process is related to membrane lipid peroxidation, membrane permeability, and the scavenging of reactive oxygen species by the antioxidant system [25].

In the present study, Zhengmai 1860, a high-yield and drought-tolerant cultivar, decreased the chlorophyll content under drought stress to a lesser extent than did drought-sensitive Zhoumai 18; this result was closely related to the ABA content in the flag leaves. Studies have shown that chlorophyll *a* and *b* can be transformed into each other [26]. The significant decrease in chlorophyll *a* content and the increase in chlorophyll *b* content in Zhengmai 1860 can be understood as the transformation of some chlorophyll *a* into chlorophyll *b*. Excessive chlorophyll *b* ensures the stability of the daylighting complex (LHC) [27]. As the *P*_n_, which is closely related to yield, has been proven to be strongly affected by drought stress [24], the lower decrease in value in Zhengmai 1860 further indicated that the senescence of Zhengmai 1860 was delayed [24]. The DE genes were also enriched in photosynthesis-related pathways, and the light-harvesting chlorophyll-binding a/b protein (LHCB) was associated with the degradation of chlorophyll under drought conditions [28]. Only the expression of *LHCB3* in Zhengmai 1860 was upregulated, which may be one of the reasons for its low degree of chlorophyll degradation.

Geranylgeranyl diphosphate reductase (CHLP) is involved in chlorophyll biosynthesis. A decrease in chlorophyll content in rice plants under stress was accompanied by a decrease in *CHLP* expression [29]. The expression correlation analysis (Supplementary Table S4) revealed that a novel miRNA (*4B_79_658524855_658524875*) may target the regulation of *CHLP* expression. This process showed marked differences after drought stress in Zhoumai 18, but there was no significant difference between Bainong 207 and Zhengmai 1860, although these negative correlations need further experimental verification (Supplementary Table S4). Fructose diphosphate aldolase (FBA) plays a role in the Calvin cycle and is affected by abiotic stress [30]. Studies have shown that the overexpression of *miR159* can affect the abundance of *FBA* under stress, but no miRNA directly acting on *FBA* has been found [31]. This study revealed that an unknown miRNA (*3B_11_22107614_22107634*) in the wheat variety Zhoumai 18 may target the *FBA*-encoding gene and downregulate its expression. Diacylglycerol kinase (DGK) can promote the production of phosphatidic acid under stress, promote ABA-mediated stomatal closure, and weaken photosynthesis [32]. In recent years, few miRNAs targeting *DGK* have been found in plants [32]. This study revealed that an unknown miRNA (*1d_104_371678191_371678211*) may target DGK, indicating that the weak photosynthetic capacity of Zhoumai 18 plants under drought conditions may be directly regulated by some unknown miRNAs.

### 3.2. miRNAs Enhance Antioxidant Capacity and Reduce the Destruction of Photosynthetic Structure

Higher antioxidant enzyme activity under drought stress can more effectively remove the reactive oxygen species produced by drought stress and reduce damage to the photosynthetic structure [33]. Chloroplast integrity is closely related to photosynthesis. Studies have shown that chloroplast ultrastructure integrity is better in drought-tolerant cultivars than in drought-sensitive cultivars [34]. After drought stress is imposed on crops, the thylakoid lamellae of the chloroplasts normally become blurred, and osmiophilic particles increase [35], which eventually causes the chloroplasts to disintegrate and release additional reactive oxygen species, accelerating leaf senescence [36]. The more stable chloroplast structure of Zhengmai 1860 showed that its tolerance to drought was better than that of Zhoumai 18, while Bainong 207 had more osmiophilic granules, and the change in thylakoid structure was not obvious, which also showed that it could tolerate drought to a certain extent. A stable photosynthetic structure is the basis of photosynthesis and further guarantees the *P*_n_. According to the transcriptome data, the expression of *CAT* and *POD* was upregulated only in Zhengmai 1860, which also proved that Zhengmai 1860 had a greater ability to scavenge ROS than the other two cultivars.

In addition, the substrate of CHLP, geranylgeranyl diphosphate, can also synthesize vitamin E [37]; the expression level of *CHLP* was decreased by *4B_79_658524855_658524875*, inhibiting the accumulation of geranylgeranyl diphosphate, which may be helpful for the synthesis of vitamin E and participation in the antioxidant process.

### 3.3. Wheat miRNAs Affect the Synthesis and Signal Transduction of Phytohormones Regulating the Synergy and Antagonism between Phytohormones

JA and ABA are both senescence-promoting phytohormones. Studies have shown that the contents of JA and ABA increase at the end of grain filling, which prevents leaves from undergoing premature senescence. However, the accumulation of ABA and JA caused the premature senescence of leaves during the early and middle grain filling stages under drought stress [38]. The interaction between JA and ABA synthesis and the crosstalk of signal transduction factors makes them closely related [8]. After drought and grain filling, the ABA content of Zhengmai 1860 decreased, and the antioxidant enzyme activity improved. In the ABA-induced MAPK pathway (Supplementary Figure S1), *MAPKKK17_18* gene expression was significantly downregulated, which also delayed the progression of leaf senescence [7]. According to the transcriptome data (Supplementary Figure S3), the expression of the *NCED*, *ABA2*, and *AAO3* genes, which are related to ABA synthesis, was upregulated, while the expression of the metabolism-related gene *CYP707A* was downregulated, which may contribute to the inhibition of ABA synthesis at the translational or posttranslational level [39].

CTK has been shown to activate drought resistance or delay leaf senescence in many plant species [40]. Moreover, the interaction between ABA and CTK can regulate leaf senescence under drought conditions [41]. In the present study, the ABA content of Zhengmai 1860 was markedly reduced, while the CTK content was markedly increased, which improved the drought resistance of Zhengmai 1860 [11]. BR was also found to promote leaf senescence [10], while the BR content of Zhengmai 1860 basically remained unchanged and that of Bainong 207 and Zhoumai 18 exhibited a marked upward trend, which was consistent with the delayed performance of leaf senescence in Zhengmai 1860. The induction of *TCH4* expression in the BR signal transduction pathway under drought stress promoted cell elongation and reduced cell division [42], while the decreased expression of *CRE1*, *B-ARR*, and *A-ARR* in the *CTK* transduction pathway also weakened cell division (Supplementary Figure S2) [43]. *A-ARR* was predicted to be the target gene of tae-miR531 (Supplementary Table S4), and its expression was negatively correlated with that of Zhoumai 18 and Zhengmai 1860, indicating that tae-miR513 may inhibit the expression of *A-ARR* during cell division under stress, thereby inhibiting cell division. The auxin response factor-encoding gene (*ARF*) is downregulated in wheat under drought stress [44]. In this study, we found that tae-miR9653b may directly regulate the expression of *ARF* and reduce its expression in Zhoumai 18 and Zhengmai 1860.

### 3.4. Mediation of TaAOS by tae-miR408 Affects the JA Content in the Flag Leaves of Wheat Plants in Response to Drought Stress

AOS is a key enzyme in the synthesis of JA that catalyzes the production of 12,13-epoxylalenic acid from 13 hydroperoxide linolenic acid, which subsequently forms the precursor of JA [45]. The decrease in JA content in the flag leaves of Zhengmai 1860 under drought conditions may be related to its regulation of leaf senescence [46]. Without affecting drought resistance, a decrease in JA content slows the decomposition of chlorophyll so that plants can better carry out photosynthesis in the middle stage before grouting [47]. Tae-miR408 has been found to target related genes and to further respond to abiotic stress by regulating the expression of target genes. In rice, miR408 targets *OsUCL8*, a plastid anthocyanin-like protein that positively regulates photosynthesis and grain yield under drought conditions [48]. Correlation analysis of the specific KEGG pathway of miRNAs in Zhengmai 1860 with the target gene showed that the *α*-linolenic acid pathway was enriched in two AOS-encoding genes and may be negatively regulated by tae-miR408. In addition, dual-luciferase reporter gene detection confirmed that tae-miR408 targets the *TaAOS* gene. The results of the enzyme-linked adsorption reaction also showed that the contents of AOS and JA in Zhengmai 1860 also decreased significantly under drought stress, and the JA content in the drought treatment group was still high and responded well to drought stress. It is speculated that tae-miR408 can regulate the senescence process of wheat flag leaves and prolong the filling time by regulating the expression of *TaAOS* and affecting the content of JA. In addition, through the analysis of transcriptome expression differences, we found that the expression of the JAZ protein [47], a transcription inhibitor in the JA signaling pathway, decreased, possibly due to widespread stress resistance regulation in wheat under drought conditions. The large accumulation of genes in the MAPK pathway and plant hormone signaling pathway also suggested that plants affect the MAPK signaling pathway by regulating the levels of phytohormones, especially ABA and JA [7].

**Figure 9:**
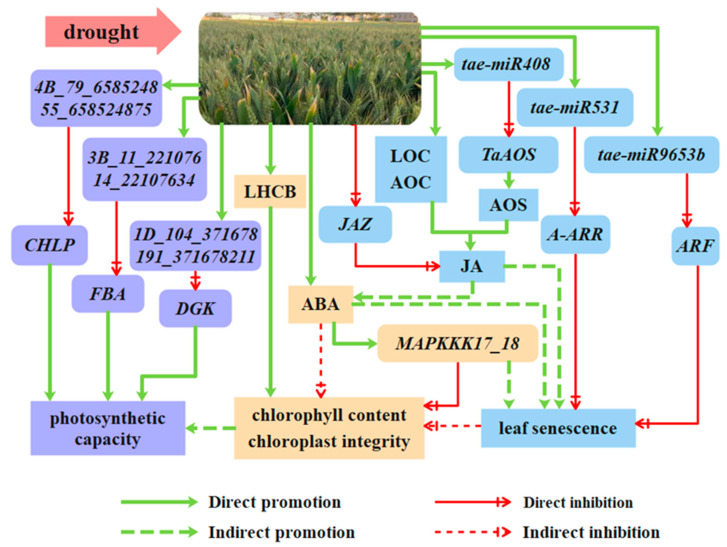
Response mechanism of flag leaves under drought stress during the grain filling period of Zhengmai 1860.

In summary, as shown in Figure 9, during drought in the early and middle stages of grain filling, Zhengmai 1860, in contrast to the strategies of Bainong 207 and Zhoumai 18, increased the expression of tae-miR408 and inhibited the translation of the *TaAOS* gene and thus reduced the content of JA and ABA. It slowed the decomposition of chlorophyll through the MAPK pathway, maintained the chloroplast structure and photosynthetic functions, and ultimately delayed leaf senescence. During this process, the content of BR did not decrease, but that of CTK became more obvious, enhancing stress resistance and inhibiting leaf cell division by regulating signal transduction and reducing the loss of energy. In addition, several novel miRNAs were revealed to be involved in regulating photosynthesis, chlorophyll synthesis, antioxidant capacity, and phytohormone signal transduction in the flag leaves of wheat plants in response to drought conditions at the grain filling stage.

## 4. Materials and Methods

### 4.1. Experimental Materials and Design

Zhengmai 1860, a new variety with high yield and drought resistance, was bred by the Wheat Research Institute of Henan Academy of Agricultural Sciences [9]. The control varieties were Bainong 207 and Zhoumai 18, which were widely planted in the main wheat production area of Huang Huai in Henan Province. Seeds of different wheat varieties of uniform size were disinfected with 5% hydrogen peroxide and soaked in water overnight. Afterwards, in the artificial climate room of the Henan Academy of Agricultural Sciences, 30 seedlings were set in each pot (divided into 3 columns, 10 plants in each column), and 10 pots were used for each variety. During the growth period, more than 70% of the maximum soil water capacity was maintained, and fertilizer was applied once at the tillering stage and once every two weeks at the jointing stage, booting stage, and filling stage, at 15 g per pot each time. The fertilizer contained potassium chloride (containing K_2_O 60%), urea (containing N 46%), and diammonium phosphate (containing P_2_O_5_ 46% and N 18%). The setting parameters of the greenhouse during the wheat heading and flowering periods were as follows: 20 °C/18 °C (day/night), a light cycle of 14 h/10 h (light/dark), a light intensity of 1000 µmol·m^−2^·s^−1^, and a relative humidity of 40%. During the filling period after flowering, the environmental temperature was 23 °C/20 °C (day/night), the photoperiod was 14 h/10 h (light/dark), the light intensity was 1200 µmol·m^−2^·s^−1^, and the relative humidity was 40%. Three wheat varieties with the same flowering time and growth vigor were identified, and the flowering dates were recorded and divided into a control (CK) group and a drought treatment (D) group [49]. In group D, watering was interrupted 15 days after flowering (pre- and mid-filling) to imitate a water shortage. At 10:00 a.m. 7 days after drought stress (22 days after flowering), the flag leaves were removed, wrapped in tin foil paper, quickly frozen in liquid nitrogen, and stored at −80 °C.

### 4.2. Determination of Chlorophyll Content

Approximately 0.1 g of leaf tissue was cut into uniform fragments and soaked in 10 mL of an acetone–ethanol mixture (the volume ratio of ethanol, acetone, and water was 4.5:4.5:1). The samples were extracted in the dark for 18 h, after which the absorbance values were measured at 663 nm and 645 nm (SpectraMax M2e, Molecular Devices, LLC, San Jose, CA, USA). The chlorophyll *a*, chlorophyll *b*, and total chlorophyll contents were calculated according to the formula of the Arnon method [50].

### 4.3. Determination of Malondialdehyde (MDA)

One gram of leaf tissue was weighed, frozen quickly in liquid nitrogen, and ground in a precooled mortar. The MDA content was calculated according to the thiobarbituric acid reactant (TBAR) [51] by measuring the absorbance at 450 nm, 532 nm, and 600 nm.

### 4.4. Determination of Superoxide Dismutase (SOD), Peroxidase (POD), and Catalase (CAT) Enzyme Activities

In accordance with Wang et al.’s method [52], the wheat flag leaves (1 g) were ground in an ice bath in 50 mM PBS (pH 7.8), after which precooled polyvinylpyrrolidone (PVP, 1%) and ethylenediaminetetraacetic acid (EDTA, 0.1 mM) were added. After the homogenized reagent was mixed by shaking at an accelerating speed, the mixture was centrifuged at a low temperature (15 min, 12,000 rpm, 4 °C), and the supernatant (extracted enzyme) was collected to determine the antioxidant enzyme activity. SOD activity was determined using the nitroblue tetrazole photochemical reduction method; CAT activity was determined using the hydrogen peroxide reduction method; and POD activity was determined using the guaiacol method. Each group was analyzed in triplicate.

### 4.5. Determination of Photosynthetic Parameters

The photosynthetic parameters of single flag leaves at the grain filling stage were measured from 9:00 a.m. to 11:00 a.m. using a Ciras-3 photosynthetic measurement system (PP systems, Amesbury, MA, USA). The leaf chamber temperature was set at 25 °C, and a light intensity of 1200 µmol·m^−2^·s^−1^ was used as the supplementary light source to measure *P*_n_, intercellular CO_2_ concentration (*C*_i_), and *g*_s_ of the wheat leaves. At least five leaves of each variety were measured.

### 4.6. Observation of Chloroplast Ultrastructure

In accordance with the previous methods used in our laboratory [53], the leaf was cut into approximately 1 mm × 3 mm pieces in the climate chamber and then prefixed in 3% glutaraldehyde in an icebox. One percent osmium tetroxide (Halin Biotechnology Co., Ltd., Shanghai, China) was used for refixation, and 30%, 50%, 70%, 90%, and 100% (100% repeated twice) acetone was used for each 12 min of gradient dehydration. The cells were embedded in Epon-812 resin. Ultrathin sections were cut on a Reichert ultramicrotome (Leica, Germany), mounted on grids, and then stained with 2% uranyl acetate and lead citrate; a HITACHI HT7700 (Hitachi, Japan) transmission electron microscope was used for observation.

### 4.7. Determination of BR, CTK, JA, and ABA Contents

Approximately 0.1 g of wheat flag leaves was ground with liquid nitrogen. The homogenate was thoroughly shaken after adding distilled water. The homogenate was used to determine the BR, CTK, JA, and ABA contents using an enzyme-linked immunosorbent assay (ELISA) kit (Shanghai Enzyme Linked Biotechnology Co., Ltd., Shanghai, China), according to the manufacturer’s recommendation. The corresponding antibody was added to each well of the enzyme-labeled plate. The phytohormone content was calculated according to the preset standard wells, and the blank wells were used as the negative control.

### 4.8. Transcriptome Sequencing

The TRIzol (Thermo Fisher Scientific Inc., Waltham, MA, USA) method was used to extract approximately 0.3 g of total RNA from the wheat flag leaves, and three biological replicates were used for quality assurance. When the extracted RNA was confirmed as complete and not decomposed, an miRNA library was established using the QIAaseq miRNA Library Kit (Qiagen, Hilden, Germany). The oligo (dT) magnetic beads and Polya were paired with A-T to enrich the mRNAs. Double-stranded cDNA was synthesized using random primer inversion. The end repair mixture and base were added to form a Y-shaped connection. The mRNA library was constructed and sequenced on an Illumina NovaSeq 6000 (USA) by cutting the gel (6% Novex TBE PAGE gel, 1.0 mm, 10 wells). The miRNA library contained 145–160 bp fragments, which were recovered by gel electrophoresis and linked to the 3′ and 5′ ends. The miRNA library was constructed using an Illumina TruSeq small RNA kit and subsequently sequenced on an Illumina 6000 platform.

### 4.9. Bioinformatics Analysis

The cardinal distribution and quality fluctuations of all the reads were determined using SeqPre (https://github.com/jstjohn/SeqPrep, accessed on 11 August 2021) and Sickle (https://github.com/najoshi/sickle, accessed on 11 August 2021) to obtain high-quality reads (Supplementary Tables S1 and S2). The HISAT2 (http://ccb.jhu.edu/software/hisat2/index.shtml, accessed on 11 August 2021) parameter was set to the default, and the sequenced data were compared with *Triticum aestivum* IWGSC RefSeq v1.0 (http://plants.ensembl.org/Triticum_aestivum/Info/Index, accessed on 11 August 2021) to perform sequence alignment. The transcriptome quality was evaluated and assembled using StringTie (http://ccb.jhu.edu/software/stringtie/, accessed on 11 August 2021), and the new transcripts were predicted and analyzed for functional annotation. RSEM (http://deweylab.github.io/RSEM/, accessed on 11 August 2021) was used to obtain the read count of each sample gene/transcript by using genome comparison results and genome annotation files. Then, TPM conversion was performed to obtain standardized gene/transcript expression levels. DeSeq2 version 1.24.0 was used to analyze the differences in expression and to screen DE genes through the use of multiples of 2.0. GO functional enrichment was analyzed using GOatools (https://github.com/tanghaibao/GOatools, accessed on 11 August 2021); KEGG pathway enrichment analysis was performed using an *R* language script; and Fisher’s exact test was used for the calculation.

The Fastx-Toolkit version 0.0.14 (http://hannonlab.cshl.edu/fastx_toolkit/, accessed on 11 August 2021) statistical method was used to calculate the base distribution of all the sequencing reads of the miRNAs and the quality fluctuations of each cycle and to screen high-quality fragments. The clean reads were compared with the reference genome sequence, as mentioned above, using Bowtie (version 1.2.3) (http://bowtie-bio.sourceforge.net/index.shtml, accessed on 11 August 2021). The readings of the reference genome were compared with those of the miRBase (http://www.mirbase.org/, accessed on 11 August 2021) and Rfam databases to obtain the annotation information of the known miRNAs. The potential new miRNAs were predicted according to the landmark hairpin structure of the precursor. The annotated sRNA sequences were aligned to the reference genome, and miReap version 0.2 (https://sourceforge.net/projects/mireap/, accessed on 11 August 2021) was used to predict the secondary structure, Dicer digestion site information, energy, and other characteristics. Finally, the sequence information, length, precursor information, and score values of the new miRNAs were obtained. The expression levels of the known miRNAs and new miRNAs in each sample were determined, and the expression levels were homogenized using TPM. After analyzing the differences in expression between the samples, psRobot v1.2 (http://omicslab.genetics.ac.cn/psRobot/, accessed on 11 August 2021) was used to predict the miRNA target genes. The target genes were also enriched according to GO and KEGG pathway analysis, and miRNA-mRNA correlation analysis was carried out to explore the possible pathways.

### 4.10. Real-Time Quantitative PCR (qRT-PCR)

Total RNA was extracted from 0.1 g of wheat flag leaves using a KK Fast Plant Total RNA Kit (centrifugal column type) (Beijing Zhuangmeng International Biogenetic Technology Co., Ltd., Beijing, China), and cDNA was synthesized using an Evo M-Mlv Reverse Transcription Premixed Kit (Aikerui Biotechnology Co., Ltd., Changsha, Hunan, China). qRT-PCR was performed using a SYBR Green Pro Taq HS Premixed qPCR Kit (Aikerui Biotechnology Co., Ltd., Changsha, Hunan, China) and a Bio-Rad iQTM5 Real-Time Fluorescent Quantitative PCR instrument (Bio-Rad Laboratories Co., Ltd., Hercules, CA, USA) according to the manufacturer’s recommendations. The miRNA was reverse transcribed to cDNA using a Poly A Tailing Kit miRNA 1st Strand cDNA Synthesis Kit (Vazyme, Nanjing, China). The expression level was determined with real-time fluorescent quantitative PCR using StepOnePlus™ (Thermo Fisher Scientific, Inc., Waltham, MA, USA). Each treatment was biologically repeated in triplicate. *β*-*Actin* was used as the internal reference gene, and the relative expression of each gene was calculated using the 2^−△△CT^ method [54]. The primers used are listed in Table 1. The primers used were designed with Primer 5 (Premier, Inc., Charlotte, NC, USA) and synthesized by Shangya Biotechnology Co., Ltd.

### 4.11. Transient Assay of miRNA-Target Interactions in Nicotiana Benthamiana Leaves

In the dual-luciferase experiment (Figure 8A), the recombinant vector pGreen0800II (0800) or *TaAOS* (0800: *TaAOS*) was mixed with the tae-miR408 overexpression vector (1300YFP: *miR408*) or the pCAMBIA1300 empty vector (1300YFP) at a ratio of 1:1. Then, we infected the plants using the *Agrobacterium tumefaciens* transformation method for 48 h. The tobacco leaves (0.05 g) were collected, and luciferase activity was detected with a TECAN multifunctional enzyme reader (Thermo Fisher Scientific, Inc., Waltham, MA, USA) using a Double Luciferase Detection Kit (Vazyme, Nanjing, China). The relative enzyme activity of each group was calculated according to the manufacturer’s recommendation.

### 4.12. Allene Oxide Synthase (AOS) Content Determination

After obtaining approximately 0.1 g of frozen wheat flag leaf homogenate, the absorbance at 450 nm was determined using an enzyme-linked immunosorbent assay (ELISA) kit (Shanghai Enzyme Linked Biotechnology Co., Ltd., Shanghai, China) according to the manufacturer’s recommendation. The AOS content was calculated according to the standard curve obtained from the standard substance.

### 4.13. Data Analysis

The treatments for each variety were repeated three times, and the data were statistically analyzed with Microsoft Office Excel 2016 (Microsoft, Redmond, WA, USA). Origin2022 (Originlab, Northampton, MA, USA) was used to conduct a one-way repeated measures ANOVA multiple comparison test and a Sidak multiple comparison post hoc test on the data (*p* < 0.05).

## Figures and Tables

**Table 1:** Primer sequences used in this study.

Gene ID or Name	Forward (5′-3′)	Reverse (5′-3′)	Application	Origin
*TraesCS5B02G124100*	GGAATCCATGAGACCACCTAC	GACCCAGACAACTCGCAAC	Internal reference	own design
*TraesCS5B02G408000*	TCTTCCACCTCCTCACCCACC	CGTCGTCGTTGTAGTTGCCG	III	own design
*TraesCS1A02G028700*	CATTGATCTCGGCAACTCTC	CTTCCCACAACTTTTCATCC	III	own design
*TraesCS5D02G207400*	ACGGGGCGGACAAGACGGAT	TGGCTCGTGTTGGAGAAGGTGA	III	own design
*TraesCS6D02G106000*	TGGTGTGGGCGGTGCT	ACGAAGGGGCCAATCTC	III	own design
*TraesCS3A02G008100*	AATGGTCTCACAATACGGGG	TGAGCCATCACCACCCAG	III	own design
*TraesCS5D02G021700*	AGTGTATCACCTAGCACCTCTC	CTCATTGGGGTAATCATCTT	III	own design
*TraesCS1D02G161700*	GTATGGGAGGTGCCTAATGG	GTCAGGATCAGAAGCGAGAA	III	own design
*TraesCS3B02G103500*	ATGAGAACCTATTTCGTCGGG	GAACAAAAGCCAATAAACCGA	III	own design
*TraesCS4B02G055600*	TAGGAGTGACCAGAGGGAAAGC	TAAGCAAATGAATCGCCGACC	III	own design
*TraesCS2B02G329400*	TTGCTATTTTTGGGTTTGGTG	AAAGATTGAACTTCTGCGAGG	III	own design
*TraesCS6A02G307700*	TCTACGGCTGGGTGTTCC	CGCCCTCGATCTTGTCCT	III	own design
*TraesCS6D02G286900*	GACTTCTACGGCTGGGTGTTCC	CCGCCCTCGATCTTATCCTTG	III	own design
*TraesCS2B02G307500*	GGAAGCCAGGGTTACAAG	GGTTTCAGCAGATTAGCG	III	own design
*TraesCS2D02G310900*	AGCCCTTAAAACGCTATCTG	TTGTCTTCCTGGTGAATCTG	III	own design
*TraesCS5D02G098800*	GTAACGGCGCAGCAGCTAAA	TTGGGACACCGACACGAAAG	III	own design
*TraesCS6B02G041900*	TGACTATCCTCTCAAGACCTCCCT	CCTTCTGCTAACCATCGCCT	III	own design
*TraesCS6B02G013300*	CAAGACCTCCCTGCTATACACG	TTCTGCTAACCATCGCCTGA	III	own design
*TraesCS5B02G408000*	agatcgccgtgtaattctagaCCTCTTCCACCTCCTCACCCA	actggtgatttcagcgaattcCTCTCCACCACCATGTCCCG	I, II	own design
*TraesCS5A02G403200*	agatcgccgtgtaattctagaTTCCACCTCCTCACCCACCG	actggtgatttcagcgaattcCCACCACCATGTCCCGCTTC	I, II	own design
1A_255_13455352_13455372	CCGCGATGGTGCTATCTTCTGGATAT		III	own design
3B_15_41449227_41449247	CCGCATCATGCCATCCTTTTGGAAG		III	own design
4D_2_6631080_6631099	CGGTAGTTCGACCGCGGAATT		III	own design
tae-miR408	CTGCACTGCCTCTTCCCTGG		III	own design
tae-miR9660-5p	CGTTGCGAGCAACGGATGAATC		III	own design
tae-miR9664-3p	CGTTGCAGTCCTCGATGTCGTAG		III	own design
4D_170_330965539_330965559	CGGTGCAATTCTCCTCTGGCATG		III	own design
7D_223_631615399_631615419	ATTATGAAGAGCGCGGGCAGC		III	own design
tae-miR408	gagaacacgggggacgagctcAGGAGGTGAGGTGGTGAACGA	gcccttgctcaccatggtaccACAGTGGCAGCATGAGAACGT	I, II	own design

Note: Gene ID refers to ensembles. Sequences of primers used for gene cloning (I), vector construction (II), and miRNA quantitative verification (III).

## Data Availability

Data Availability Statement: Raw data were deposited in the NCBI Short Read Archive (SRA) database, and the accession numbers are PRJNA1077678 and PRJNA1077667.

## Supplementary Materials

The supporting information can be downloaded at: https://www.mdpi.com/article/10.3390/ijms25105518/s1.
