# Supplementary material for: Differential Transcription Profiling Reveals the MicroRNAs Involved in Alleviating Damage to Photosynthesis under Drought Stress during the Grain Filling Stage in Wheat
Source: Int J Mol Sci. 2024 May 18;25(10):5518. doi: 10.3390/ijms25105518 (PMC11122533; doi:10.3390/ijms25105518)
Supplement: Supplementary file 1 [file ijms-25-05518-s001.zip › Supplementary Figure S3.pdf]

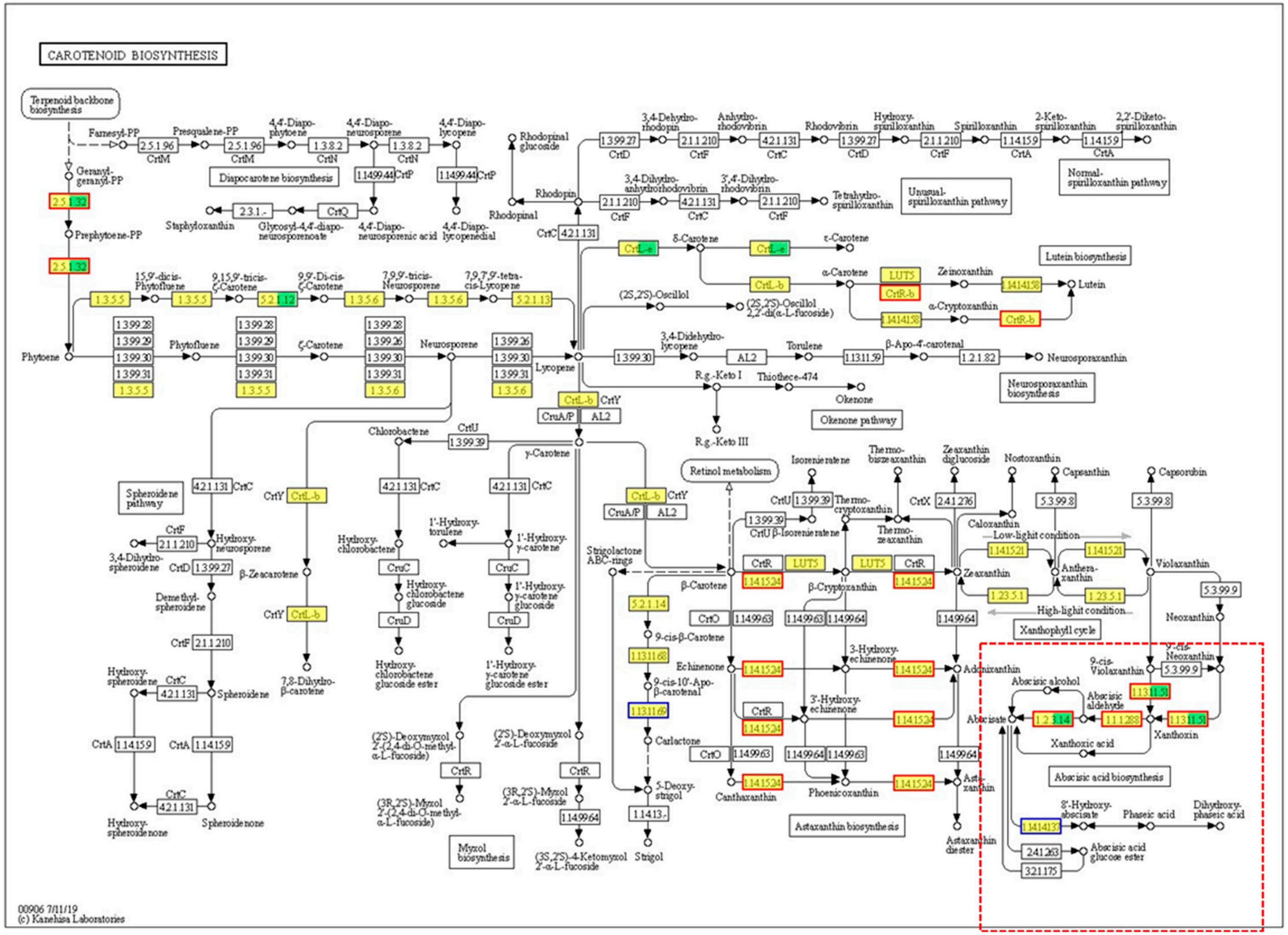

Fig. S3 KEGG enrichment map of the ABA synthesis pathway in Zhengmai 1860 DE Gene. The red border represents upregulated genes, while the green border represents downregulated genes.
