# Supplementary material for: Differential Transcription Profiling Reveals the MicroRNAs Involved in Alleviating Damage to Photosynthesis under Drought Stress during the Grain Filling Stage in Wheat
Source: Int J Mol Sci. 2024 May 18;25(10):5518. doi: 10.3390/ijms25105518 (PMC11122533; doi:10.3390/ijms25105518)
Supplement: Supplementary file 1 [file ijms-25-05518-s001.zip › Supplementary Table S1.pdf]

Table S1 Statistical table of miRNA quality control data

Sample	Raw reads	Raw bases	Clean reads	Clean bases	Error rate(%)	Q20(%)	Q30(%)	GC content(%)	Useful reads(18nt-32nt)
CK1_18	13233081	992481075	12164696	252633109	0.0225	99.05	96.82	44.25	8943092
CK2_18	17736096	1330207200	17062293	378635560	0.0224	99.06	96.87	44.24	14032579
CK3_18	15995843	1199688225	15257649	337415426	0.0227	98.9	96.53	44.44	12255478
CK1_1860	16263686	1219776450	15276527	318744541	0.0225	99.02	96.78	45.97	10601575
CK2_1860	17031771	1277382825	16048783	325099019	0.0223	99.11	96.97	47.17	10526935
CK3_1860	14883417	1116256275	14052264	297992409	0.0226	99.02	96.69	46.52	9672986
CK1_207	14510058	1088254350	13647610	294843594	0.0224	99.07	96.91	45.25	10380253
CK2_207	15642639	1173197925	14938980	326180302	0.0225	99.04	96.71	46.37	11226411
CK3_207	13510101	1013257575	12887994	284186127	0.0227	98.94	96.59	46.12	9503529
D1_18	18990786	1424308950	17955874	380108423	0.0223	99.13	97	47.39	13264168
D2_18	11666528	874989600	10931830	242666418	0.0224	99.07	96.91	50.99	7843292
D3_18	16115970	1208697750	15792352	304763440	0.0222	99.12	97.15	55.83	10699101
D1_1860	18490435	1386782625	17129668	333201597	0.0228	98.88	96.41	50.51	9215233
D2_1860	17431091	1307331825	16162447	333524892	0.0226	98.99	96.61	49.31	10603073
D3_1860	17738487	1330386525	16461952	330877408	0.0227	98.95	96.56	48.33	10595333
D1_207	14691821	1101886575	14028929	305552285	0.0225	99.04	96.81	47.57	9914225
D2_207	17075676	1280675700	15881160	341113184	0.0225	99.02	96.81	46.17	11378834
D3_207	16048829	1203662175	15241115	331560568	0.0226	98.99	96.62	47.36	10589491
