# Supplementary material for: Differential Transcription Profiling Reveals the MicroRNAs Involved in Alleviating Damage to Photosynthesis under Drought Stress during the Grain Filling Stage in Wheat
Source: Int J Mol Sci. 2024 May 18;25(10):5518. doi: 10.3390/ijms25105518 (PMC11122533; doi:10.3390/ijms25105518)
Supplement: Supplementary file 1 [file ijms-25-05518-s001.zip › Supplementary Table S2.pdf]

Table S2 Statistical table of mRNA quality control data

Sample	Raw reads	Raw bases	Clean reads	Clean bases	Error rate(%)	Q20(%)	Q30(%)	GC content(%)
CK1_18	57175612	8633517412	56262982	8355533825	0.0255	97.73	93.73	55.94
CK2_18	60373144	9116344744	59612826	8851426225	0.0256	97.71	93.68	55.23
CK3_18	65140894	9836274994	64472706	9600053228	0.0256	97.71	93.65	55.6
CK1_1860	62470854	9433098954	61698822	9134819544	0.0255	97.74	93.75	55.46
CK2_1860	59955562	9053289862	59238698	8775525290	0.0255	97.77	93.78	55.22
CK3_1860	61316924	9258855524	60529888	8985397681	0.0256	97.71	93.68	55.48
CK1_207	63994396	9663153796	63262208	9373844599	0.0254	97.78	93.81	55.42
CK2_207	57778196	8724507596	56983016	8456253595	0.0254	97.8	93.86	54.47
CK3_207	56184586	8483872486	55344446	8230683291	0.0256	97.71	93.64	54.45
D1_18	68637916	10364325316	67763680	10036049052	0.0258	97.63	93.47	54.69
D2_18	60231538	9094962238	59365982	8789976018	0.0254	97.8	93.86	54.75
D3_18	54729294	8264123394	53698924	8008773420	0.0255	97.76	93.77	53.13
D1_1860	56999924	8606988524	56124888	8297878808	0.0255	97.73	93.72	54.98
D2_1860	57773890	8723857390	57031348	8470418762	0.0255	97.76	93.77	55.37
D3_1860	55572700	8391477700	54849626	8127471959	0.0253	97.85	93.95	54.97
D1_207	58894660	8893093660	58197076	8664336180	0.0255	97.75	93.76	55.49
D2_207	57634404	8702795004	56823152	8473749304	0.0255	97.73	93.71	54.85
D3_207	57493620	8681536620	56743080	8440666810	0.0257	97.67	93.55	55.03
