# Supplementary material for: Differential Transcription Profiling Reveals the MicroRNAs Involved in Alleviating Damage to Photosynthesis under Drought Stress during the Grain Filling Stage in Wheat
Source: Int J Mol Sci. 2024 May 18;25(10):5518. doi: 10.3390/ijms25105518 (PMC11122533; doi:10.3390/ijms25105518)
Supplement: Supplementary file 1 [file ijms-25-05518-s001.zip › Supplementary Table S3.pdf]

Table S3 Details of predicted new miRNAs

miRNA name	miRNA seq	miRNA len	pre-miRNA name	pre-miRNA len	pre position	energy
1A_104_419025529_419025550	CGAATGTATTTTTTATGGCTTG	22	1A_104	78	1A(+):419025473-419025550	-77.9
1A_108_480678204_480678223	TGAGAAGGTATATCATAATT	20	1A_108	172	1A(+):480678052-480678223	-54.2
1A_119_541706924_541706944	AGAGCGCGCCGCCGTCGAGGG	21	1A_119	50	1A(+):541706895-541706944	-102.5
1A_140_588909739_588909760	CATGGCCAAGGCCTCTGAGGTC	22	1A_140	50	1A(-):588909711-588909760	-97.1
1A_188_477181577_477181597	TGCCTAGATCCACACTCATCC	21	1A_188	277	1A(-):477181321-477181597	-62.2
1A_190_474709464_474709483	TGAGTAGGCCACGGGAGGCA	20	1A_190	72	1A(-):474709412-474709483	-93.8
1A_195_441303094_441303113	ATCAAGGAAATGGCAAGGGT	20	1A_195	175	1A(-):441303094-441303268	-71.5
1A_216_280495408_280495427	TGAGTAGGCCACGGGAGACG	20	1A_216	72	1A(-):280495356-280495427	-106
1A_225_147048367_147048386	GATAGATGATTTGTGGCATT	20	1A_225	104	1A(-):147048367-147048470	-53.5
1A_255_13455352_13455372	ATGGTGCTATCTTCTGGATAT	21	1A_255	67	1A(-):13455306-13455372	-68.8
1A_34_49996946_49996967	TCAGATGAGAAGGCAGATCATA	22	1A_34	211	1A(+):49996757-49996967	-81.8
1A_55_79167066_79167086	GAAACGTTGGATGGTTGTGGC	21	1A_55	201	1A(+):79167066-79167266	-113.7
1A_82_326329279_326329299	CTCGGACCAGGCTTCATTCCC	21	1A_82	94	1A(+):326329206-326329299	-121
1A_96_417395368_417395388	TGGAAACGCCCGCACAAACTC	21	1A_96	47	1A(+):417395342-417395388	-94.8
1B_207_674031137_674031158	TTTTGCTGGTTGAACAACCTCA	22	1B_207	107	1B(-):674031052-674031158	-89.7
1B_222_638439242_638439262	GGGGAAATAAGGAGTAACGAC	21	1B_222	65	1B(-):638439242-638439306	-117.3
1B_244_543593556_543593575	CAAAGCATCTTGAGGGACCA	20	1B_244	182	1B(-):543593556-543593737	-143.6
1B_269_437834565_437834586	CGAATGTATTTTTTATGGCTTG	22	1B_269	78	1B(-):437834565-437834642	-72

miRNA name	miRNA seq	miRNA len	pre-miRNA name	pre-miRNA len	pre position	energy
1B_276_403978995_403979014	TGAGTAGGCCACGGGAGGCA	20	1B_276	72	1B(-):403978943-403979014	-102.4
1B_281_357916236_357916256	CTCGGACCAGGCTTCATTCCC	21	1B_281	94	1B(-):357916236-357916329	-117.2
1B_300_63962818_63962838	TGTTATGATATGATCATTGAT	21	1B_300	209	1B(-):63962630-63962838	-63.1
1B_35_77543987_77544008	TTAGATGAGAAGGCAGATCATA	22	1B_35	203	1B(+):77543806-77544008	-93
1B_54_106903718_106903738	ATGTATCACTGTAGAGGCGGA	21	1B_54	163	1B(+):106903718-106903880	-107.1
1B_56_107369352_107369372	TTCGCCGGAGCAGCGTGCTGT	21	1B_56	53	1B(+):107369352-107369404	-141.2
1B_97_392753730_392753750	TCTTCGCCGGCTGCGTGTTCC	21	1B_97	53	1B(+):392753730-392753782	-129.7
1D_104_371678191_371678211	TAAGAATATTCTTTATCTGTT	21	1D_104	262	1D(+):371677950-371678211	-69
1D_121_423763005_423763025	ATTCTTTTTGCTGGAACCGGC	21	1D_121	227	1D(+):423763005-423763231	-97.8
1D_183_375963504_375963523	TGAGTAGGCCACGGGAGGCA	20	1D_183	72	1D(-):375963452-375963523	-91.6
1D_193_325491978_325491999	CGAATGTATTTTTTATGGCTTG	22	1D_193	78	1D(-):325491978-325492055	-76.5
1D_195_322381310_322381330	TGGAAACGCCCGCACAAACTC	21	1D_195	47	1D(-):322381310-322381356	-130.9
1D_197_306632526_306632546	TCCATGGGTGTTTTGATCATT	21	1D_197	189	1D(-):306632526-306632714	-98.8
1D_198_299762025_299762044	TGAGTAGGCCACGGGAGGCA	20	1D_198	72	1D(-):299761973-299762044	-117.6
1D_210_203139676_203139696	AAAAATACAGACGGCAAACAA	21	1D_210	128	1D(-):203139569-203139696	-106
1D_231_13504834_13504853	CTTGAGAGACCAAAATCAGC	20	1D_231	43	1D(-):13504834-13504876	-60.1
1D_32_50960270_50960291	TCAGATGAGAAGGCAGATCATA	22	1D_32	201	1D(+):50960091-50960291	-96.3
1D_59_59869980_59870000	AAAGGAATATGTGAGGGCTTA	21	1D_59	182	1D(+):59869819-59870000	-60.1
1D_63_85179603_85179623	GAAACGTTGGATGGTTGTGGC	21	1D_63	158	1D(+):85179466-85179623	-60.9

miRNA name	miRNA seq	miRNA len	pre-miRNA name	pre-miRNA len	pre position	energy
1D_77_254662315_254662335	CTCGGACCAGGCTTCATTCCC	21	1D_77	94	1D(+):254662242-254662335	-121.6
2A_119_559072377_559072397	AGGTGCAGTGGCACATGCAGC	21	2A_119	85	2A(+):559072313-559072397	-100
2A_128_593354784_593354804	TTAGATGACCATCAGCAAACA	21	2A_128	137	2A(+):593354668-593354804	-125.3
2A_158_734900513_734900533	TCCATGGGTATTTTGATCATT	21	2A_158	275	2A(+):734900513-734900787	-55.3
2A_160_737476951_737476971	TCCGCGATCATCATGACCAAA	21	2A_160	196	2A(+):737476951-737477146	-93
2A_183_765572886_765572906	TCCATGTGTATTTTGATCATT	21	2A_183	169	2A(+):765572738-765572906	-63.1
2A_193_778725883_778725903	TCCATGGGTATTTTGGTCATT	21	2A_193	198	2A(-):778725883-778726080	-116.1
2A_198_774802453_774802473	TTCCAAAGGGATCGCATTGAT	21	2A_198	78	2A(-):774802396-774802473	-109.7
2A_219_711074211_711074230	GATGACAATATGATGCTCAA	20	2A_219	43	2A(-):711074188-711074230	-122.6
2A_266_185964095_185964115	AATTTGCACGGAAGTAGATTG	21	2A_266	179	2A(-):185964095-185964273	-84.3
2A_271_149238449_149238469	TTGGTGTCACCTCGCCTGAAC	21	2A_271	91	2A(-):149238449-149238539	-135.7
2A_275_149235042_149235062	TTGGTGTCACCTCGCCTGAAC	21	2A_275	82	2A(-):149235042-149235123	-143.1
2A_280_132860679_132860699	TTCATTTGGTTGAATTGGGGA	21	2A_280	78	2A(-):132860679-132860756	-108.3
2A_284_102126969_102126989	TGAAGTGGCGATTCTGGAAGA	21	2A_284	99	2A(-):102126969-102127067	-105.1
2A_67_137567719_137567739	TCCATGGGTATTTTGGTCATT	21	2A_67	197	2A(+):137567543-137567739	-97.3
2A_74_179565254_179565274	TTCCAAAATCGCCACTCCATA	21	2A_74	99	2A(+):179565254-179565352	-101.1
2B_0_684549_684570	TGCTTGAAGCCATTGGAATTTT	22	2B_0	268	2B(+):684549-684816	-80.8
2B_112_528817823_528817843	TTAGATGACCATCAGCAAACA	21	2B_112	122	2B(+):528817722-528817843	-123.4
2B_136_736890116_736890136	TCCGCGATCATCACGACCAAA	21	2B_136	98	2B(+):736890039-736890136	-107

miRNA name	miRNA seq	miRNA len	pre-miRNA name	pre-miRNA len	pre position	energy
2B_155_754964234_754964253	AAGTCCCTCTATCCCCACCT	20	2B_155	158	2B(+):754964096-754964253	-85.4
2B_164_786139783_786139803	TTCCAAAGGGATCGCATTGAT	21	2B_164	69	2B(+):786139783-786139851	-62.2
2B_181_793422976_793422996	TGATAGATGACATGTGGCATT	21	2B_181	201	2B(-):793422796-793422996	-89.32
2B_196_748346185_748346204	TGAAGTGTTTGGAGGAACTC	20	2B_196	268	2B(-):748345937-748346204	-109.9
2B_198_748084356_748084375	TGAAGTGTTTGGAGGAACTC	20	2B_198	125	2B(-):748084251-748084375	-106.4
2B_203_732048718_732048738	TCATCTGGCACTGCTTTCTCT	21	2B_203	259	2B(-):732048718-732048976	-99.7
2B_205_715301167_715301187	TCCATGGGTATTTTGGTCATT	21	2B_205	196	2B(-):715301167-715301362	-95.7
2B_223_638503113_638503133	TCCATGGGTATTTTGGTCATT	21	2B_223	158	2B(-):638503113-638503270	-82.9
2B_269_180042064_180042084	TTCATTTGGTTGAATTAGGGA	21	2B_269	78	2B(-):180042064-180042141	-100
2B_270_174800031_174800051	TCTTCGCCGGCTGCGCGTTCC	21	2B_270	53	2B(-):174799999-174800051	-136.9
2B_273_158881603_158881623	TGAGCGCACCGCCGTCGAGGG	21	2B_273	50	2B(-):158881603-158881652	-141
2B_278_138630730_138630750	TCCCAAAATCGCCACTCCATA	21	2B_278	98	2B(-):138630653-138630750	-143.3
2B_36_37708929_37708949	TCAAATCAAGGACCATATCAT	21	2B_36	137	2B(+):37708813-37708949	-65.8
2B_58_75239487_75239507	AAGGGCAAGGAGCTCCGCCGA	21	2B_58	158	2B(+):75239350-75239507	-123.2
2B_5_5124766_5124786	AATTTGCACGGAAGTAGATTG	21	2B_5	181	2B(+):5124606-5124786	-84.97
2B_77_210255000_210255020	TTGGTGTCACCTCGCCTGAAC	21	2B_77	91	2B(+):210254930-210255020	-132
2B_81_210258124_210258144	ATCAGGAGAGATGACACCGAC	21	2B_81	93	2B(+):210258124-210258216	-132.6
2D_101_601696032_601696052	TCCATGGGTATTTTGATCATT	21	2D_101	276	2D(+):601696032-601696307	-58.6
2D_138_650221339_650221359	TTCCAAAGGGATCGCATTGAT	21	2D_138	78	2D(+):650221339-650221416	-112.2

miRNA name	miRNA seq	miRNA len	pre-miRNA name	pre-miRNA len	pre position	energy
2D_161_613024157_613024176	TGAAGTGTTTGGAGGAACTC	20	2D_161	263	2D(-):613023914-613024176	-121.6
2D_164_613009694_613009713	TGAAGTGTTTGGAGGAACTC	20	2D_164	64	2D(-):613009694-613009757	-82.5
2D_165_612998307_612998326	TGAAGTGTTTGGAGAAACTC	20	2D_165	54	2D(-):612998273-612998326	-111.2
2D_167_572791673_572791693	TGAAGTGGCGATTCTAGAAAA	21	2D_167	253	2D(-):572791441-572791693	-60.8
2D_169_572078658_572078677	GATGACAATATGATGCTCAA	20	2D_169	43	2D(-):572078635-572078677	-104.3
2D_187_449570580_449570600	TTAGATGACCATCAGCAAACA	21	2D_187	123	2D(-):449570580-449570702	-115.4
2D_234_126126279_126126299	TTCATTTGGTTGAATTAGGGA	21	2D_234	78	2D(-):126126279-126126356	-98.9
2D_247_43951450_43951470	TCTCTGGCGGTAGGACTTACC	21	2D_247	69	2D(-):43951402-43951470	-101.3
2D_40_151985179_151985199	TTGGTGTCACCTCGCCTGAAC	21	2D_40	95	2D(+):151985105-151985199	-140.6
2D_44_152018586_152018606	TTGGTGTCACCTCGCCTGAAC	21	2D_44	91	2D(+):152018516-152018606	-131.7
2D_62_326455651_326455671	ATCCAAAGGGATCGCATTGAT	21	2D_62	69	2D(+):326455651-326455719	-88.7
2D_75_451153847_451153867	TGAAGTGGCGATTCTGGAAAA	21	2D_75	99	2D(+):451153769-451153867	-111
3A_119_443867389_443867409	ATTTTGGGCAAGTAAGTTATT	21	3A_119	89	3A(+):443867321-443867409	-89
3A_142_638710274_638710294	TCCATGGGTATTTTGATCATG	21	3A_142	198	3A(+):638710097-638710294	-90.4
3A_151_658833104_658833124	TTAATTTGTCCATAGCATCAG	21	3A_151	95	3A(+):658833104-658833198	-98.1
3A_16_33150703_33150724	TTCGCCGGTGGCACATTTCCCT	22	3A_16	48	3A(+):33150703-33150750	-134.2
3A_192_711072176_711072197	TAATCTTTTGGAAATATGCTTA	22	3A_192	234	3A(+):711072176-711072409	-72.3
3A_207_737797813_737797833	ATTGTTGGATGATAATTTGGT	21	3A_207	93	3A(+):737797813-737797905	-65.8
3A_260_709405806_709405826	TCTATGGGTATTTTGATCATT	21	3A_260	198	3A(-):709405806-709406003	-97.4

miRNA name	miRNA seq	miRNA len	pre-miRNA name	pre-miRNA len	pre position	energy
3A_262_704459003_704459024	ATAAGCACCGATGCTTAGAGAA	22	3A_262	84	3A(-):704459003-704459086	-118.6
3A_270_665987256_665987276	TGAAGTGGCGATTCTGGAAAA	21	3A_270	99	3A(-):665987256-665987354	-134.2
3A_28_76564146_76564167	GTTGACAGAAGAGAGTGAGCAC	22	3A_28	93	3A(+):76564146-76564238	-137.3
3A_294_463121605_463121625	CTTCAGAAGAGCATGTCAGTT	21	3A_294	243	3A(-):463121605-463121847	-82.4
3A_297_428795149_428795169	TGGAGTGGCGATTCTGGAAAA	21	3A_297	218	3A(-):428794952-428795169	-77
3A_300_373156089_373156109	ATGTAGAAGCACCAGGGTAAG	21	3A_300	80	3A(-):373156089-373156168	-90.9
3A_395_33260416_33260436	ATCCAAAGGGATCGCATTGAT	21	3A_395	92	3A(-):33260345-33260436	-95.9
3B_11_22107614_22107634	TACCCTCGCCGGAGTAGCGTG	21	3B_11	61	3B(+):22107614-22107674	-171.8
3B_125_486007487_486007507	TGATAGATGACATGTGGCATT	21	3B_125	117	3B(+):486007487-486007603	-148.3
3B_13_41449157_41449177	ATCCAAAGGGATCGCATTGAT	21	3B_13	92	3B(+):41449157-41449248	-100.3
3B_151_625190881_625190901	CTGGACGATTCAGAGACGATA	21	3B_151	102	3B(+):625190881-625190982	-99.4
3B_157_691444835_691444855	TTAATTTGTCCATAGCATCCG	21	3B_157	100	3B(+):691444835-691444934	-99.6
3B_15_41449227_41449247	ATCATGCGATCCTTTTGGAAG	21	3B_15	144	3B(+):41449227-41449370	-72.2
3B_169_748321324_748321343	GAGTCCCGGGCAACCCACGA	20	3B_169	85	3B(+):748321324-748321408	-124.8
3B_210_766020388_766020409	TAATCTTTTGGAAATATGCTTA	22	3B_210	232	3B(+):766020388-766020619	-67.9
3B_23_89094488_89094507	TAGGTGACTTTGGTCTCTCA	20	3B_23	85	3B(+):89094488-89094572	-96.6
3B_283_794580798_794580818	CTTCATAAGAGCATGTCATTT	21	3B_283	80	3B(-):794580739-794580818	-99.7
3B_332_689011496_689011516	TTCGCCGGAGCAGCGTGCTGT	21	3B_332	53	3B(-):689011464-689011516	-138.1
3B_346_595799659_595799678	TTGAGGTCTAAGTGGAGAAA	20	3B_346	121	3B(-):595799558-595799678	-93.6

miRNA name	miRNA seq	miRNA len	pre-miRNA name	pre-miRNA len	pre position	energy
3B_352_568258924_568258944	ACATCTCTGAAGTTGACACCA	21	3B_352	243	3B(-):568258924-568259166	-67.5
3B_368_464356310_464356330	GGAATCCTGTCACCTTGACCA	21	3B_368	203	3B(-):464356310-464356512	-68.2
3B_377_247570409_247570429	AGTTGAAGATGAGATATTGAA	21	3B_377	65	3B(-):247570365-247570429	-99.2
3B_432_109363904_109363925	GTTGACAGAAGAGAGTGAGCAC	22	3B_432	93	3B(-):109363833-109363925	-131.8
3B_436_89352830_89352849	TAGGTGACTTTGGTCTCTCA	20	3B_436	93	3B(-):89352757-89352849	-83.1
3B_44_128797155_128797175	AAATTTTCCATGGCAAATGTA	21	3B_44	144	3B(+):128797032-128797175	-71.9
3B_9_16893194_16893214	TGATGGATGATATGTGGCATT	21	3B_9	119	3B(+):16893194-16893312	-148
3D_125_583160591_583160611	TCAGTGGCAAACTCATCTGCA	21	3D_125	66	3D(+):583160546-583160611	-78.7
3D_152_605340217_605340236	CAAAGCATCTTGACGGACCA	20	3D_152	89	3D(-):605340217-605340305	-74.2
3D_17_113564010_113564029	TGAGTAGGCCACGGGAGGCA	20	3D_17	73	3D(+):113564010-113564082	-92.7
3D_202_573769625_573769645	CCGCGTCGCCGTCTCCCGCTC	21	3D_202	240	3D(-):573769625-573769864	-76.3
3D_209_544447561_544447581	TGAGATTTCCATACTGTGGGC	21	3D_209	91	3D(-):544447491-544447581	-91.2
3D_211_524220888_524220908	TTAATTTGTCCATAGCATCCG	21	3D_211	100	3D(-):524220809-524220908	-90.7
3D_213_523446498_523446518	AGGGTCGAACTGAGAACACAT	21	3D_213	70	3D(-):523446449-523446518	-102
3D_220_513889505_513889525	TTGGGTGTGCATGATATTACT	21	3D_220	274	3D(-):513889505-513889778	-52.4
3D_233_453160545_453160565	CCTTCGCCGGCTGCGCGTTCC	21	3D_233	53	3D(-):453160513-453160565	-151.6
3D_292_65610352_65610373	GTTGACAGAAGAGAGTGAGCAC	22	3D_292	93	3D(-):65610281-65610373	-146.9
3D_308_23964697_23964717	ATCCAAAGGGATCGCATTGAT	21	3D_308	92	3D(-):23964626-23964717	-105.6
3D_48_143964743_143964763	TACGGCCTGATGACATCCACA	21	3D_48	101	3D(+):143964663-143964763	-105.1

miRNA name	miRNA seq	miRNA len	pre-miRNA name	pre-miRNA len	pre position	energy
3D_55_185433288_185433308	TGTTTGGATTTTGAGGTATGT	21	3D_55	43	3D(+):185433288-185433330	-61.1
3D_95_524000761_524000781	TTAATTTGTCCATAGCATCCG	21	3D_95	100	3D(+):524000761-524000860	-98.4
4A_139_674762546_674762567	TTTTGCTGGTTGAACGACCTCA	22	4A_139	102	4A(+):674762546-674762647	-118.4
4A_195_736518941_736518961	GGTTGATGAGGTGAATTTGAT	21	4A_195	221	4A(+):736518741-736518961	-73.4
4A_214_743552709_743552729	TGATAGATGACATGTGGCATT	21	4A_214	118	4A(-):743552612-743552729	-92.1
4A_332_628471095_628471115	ACGTCCACCGACACCATGAAG	21	4A_332	152	4A(-):628471095-628471246	-96.4
4A_386_248805616_248805635	AAGAAGCAAATCTTGAACAG	20	4A_386	91	4A(-):248805616-248805706	-71.9
4A_411_104238549_104238569	GGGAAAAGAAGGAGTAACGAC	21	4A_411	65	4A(-):104238549-104238613	-110.9
4A_431_16475407_16475427	TTCGGACCAGGCTTCATTCCC	21	4A_431	95	4A(-):16475407-16475501	-100.3
4A_82_580566457_580566476	GTAAAAAATTGCGGATTTTT	20	4A_82	173	4A(+):580566304-580566476	-86.5
4A_91_609396137_609396157	TAATCTCACCTCAACAGCCGC	21	4A_91	86	4A(+):609396072-609396157	-107
4A_96_609462193_609462213	TAATCTCAACTCAACAGCCGC	21	4A_96	90	4A(+):609462124-609462213	-112.4
4B_0_50724622_50724642	TTAGGACTGGAGATGAACAAA	21	4B_0	63	4B(+):50724622-50724684	-52.3
4B_20_299561392_299561412	AAAAATGGCCCGGCCTGTAAA	21	4B_20	200	4B(+):299561213-299561412	-176
4B_65_613386241_613386261	TGGAAGGGGCATGCAGAGGAG	21	4B_65	86	4B(+):613386241-613386326	-114
4B_79_658524855_658524875	TACCCTCGCCGGAGCAGCGTG	21	4B_79	61	4B(-):658524815-658524875	-164.6
4D_101_483166529_483166549	TGGAAGGGGCATGCAGAGGAG	21	4D_101	86	4D(+):483166529-483166614	-114.8
4D_104_487368507_487368527	TTTGGGGGAGATTTTTTTGCG	21	4D_104	48	4D(+):487368480-487368527	-88.8
4D_111_507462048_507462068	CTTGAACTTCTCCATAGCATC	21	4D_111	104	4D(+):507462048-507462151	-96.5

miRNA name	miRNA seq	miRNA len	pre-miRNA name	pre-miRNA len	pre position	energy
4D_115_507476556_507476576	CTTGAACTTCTCCATAGCATC	21	4D_115	104	4D(+):507476556-507476659	-97.9
4D_165_369667739_369667760	ATAAGCACCGATACTTAAAGAA	22	4D_165	86	4D(-):369667739-369667824	-105.7
4D_170_330965539_330965559	GTGCAATTCTCCTCTGGCATG	21	4D_170	91	4D(-):330965469-330965559	-90.3
4D_205_70592532_70592552	AGAGCGCACCGCCGTCGAGGG	21	4D_205	52	4D(-):70592532-70592583	-153.6
4D_207_70586230_70586250	AGAGCGCACCGCCGTCGAGGG	21	4D_207	52	4D(-):70586230-70586281	-151.2
4D_224_10922291_10922311	CCTTCGCCGGCTGCGCGTTCC	21	4D_224	53	4D(-):10922259-10922311	-132.6
4D_29_185923527_185923548	TAATGAGTTTTTTTTGATTGTT	22	4D_29	266	4D(+):185923527-185923792	-52.6
4D_2_6631080_6631099	GGTAGTTCGACCGCGGAATT	20	4D_2	264	4D(+):6631080-6631343	-73.4
4D_36_318329026_318329046	TGAGAATATTCTTTATCTGTT	21	4D_36	262	4D(+):318328785-318329046	-86
4D_41_347521630_347521649	TGAGTAGGCCACGGGAGGCA	20	4D_41	70	4D(+):347521630-347521699	-78.6
4D_79_450196378_450196398	TTCGGACCAGGCTTCATTCCC	21	4D_79	88	4D(+):450196311-450196398	-103.1
5A_118_663320640_663320660	TGGAAGGGGCATGCAGAGGAG	21	5A_118	83	5A(+):663320640-663320722	-118.9
5A_132_691798483_691798503	TGAATTTGAACATAGCATCAG	21	5A_132	93	5A(-):691798411-691798503	-117.8
5A_148_653058480_653058500	CAATCTCACCTCAACAGCCGC	21	5A_148	86	5A(-):653058480-653058565	-79.3
5A_167_578021263_578021283	TTCGGACCAGGCTTCATTCCC	21	5A_167	70	5A(-):578021263-578021332	-59.7
5A_198_505603379_505603400	GGAGTGTCACTGAGAACACAAG	22	5A_198	84	5A(-):505603317-505603400	-102.9
5A_202_505600511_505600532	GGAGTGCCACTGAGAACACAAG	22	5A_202	82	5A(-):505600451-505600532	-113.5
5A_21_116585637_116585657	TCAAGAATCATATCATGGACA	21	5A_21	151	5A(+):116585507-116585657	-72.2
5A_259_298549535_298549555	TTCGCCGGAGCAGCGTGCAGA	21	5A_259	52	5A(-):298549504-298549555	-167.9

miRNA name	miRNA seq	miRNA len	pre-miRNA name	pre-miRNA len	pre position	energy
5A_270_186739728_186739748	GGGAAAAGAAGGAGTAACGAC	21	5A_270	65	5A(-):186739728-186739792	-103.9
5A_283_78451891_78451911	TTGAGACGAACACAGACCAAC	21	5A_283	92	5A(-):78451891-78451982	-122
5A_289_53588819_53588839	AGGTCATGCTGGAGTTTCATC	21	5A_289	77	5A(-):53588819-53588895	-107.7
5A_2_3385877_3385897	TTGTTGGATGATAATATGTTA	21	5A_2	51	5A(+):3385877-3385927	-60.6
5A_36_194712553_194712573	TGATAGATGACATGTGGCATT	21	5A_36	126	5A(+):194712553-194712678	-176.1
5A_54_322477297_322477317	TTTGGATTGAAGGGATTTTAT	21	5A_54	116	5A(+):322477202-322477317	-62.3
5A_62_446358829_446358849	GGGAAAAGAAGGAGTAACGAC	21	5A_62	65	5A(+):446358785-446358849	-101.2
5A_63_461486150_461486170	TGATGGATGATATGTGGCATT	21	5A_63	61	5A(+):461486150-461486210	-101.3
5A_80_494429966_494429985	TGCTCTCTTGGAACAACCTC	20	5A_80	44	5A(+):494429942-494429985	-89.6
5A_8_10846730_10846751	ATAAGCACCGAAACTTAGAGAA	22	5A_8	53	5A(+):10846730-10846782	-62.1
5A_95_572017038_572017058	CCTTCGCCGGCTGCGCGTTCC	21	5A_95	53	5A(+):572017038-572017090	-117.8
5B_103_441838371_441838391	TGAGTTGGATTATGTCAAGCA	21	5B_103	225	5B(+):441838371-441838595	-95.7
5B_111_477514109_477514129	ATTGCTGGATGATAATATGGT	21	5B_111	267	5B(+):477514109-477514375	-71.8
5B_131_608863022_608863042	CCCTGATGGATGATATGTGGC	21	5B_131	128	5B(+):608863022-608863149	-140.8
5B_180_711071047_711071067	TAATCTCAACTCAACAGCCGC	21	5B_180	90	5B(+):711070978-711071067	-82.9
5B_189_705495211_705495231	TAATCTTACCTCAACAGCCGC	21	5B_189	90	5B(-):705495211-705495300	-93.4
5B_253_583164381_583164401	TCCATAGGTATTTTGATCATT	21	5B_253	194	5B(-):583164381-583164574	-92.9
5B_256_562984083_562984103	TTCGGACCAGGCTTCATTCCC	21	5B_256	74	5B(-):562984083-562984156	-64.7
5B_273_480241534_480241555	GGAGTGCCTCTGAGAACACAAG	22	5B_273	82	5B(-):480241474-480241555	-116.7

miRNA name	miRNA seq	miRNA len	pre-miRNA name	pre-miRNA len	pre position	energy
5B_307_398345215_398345235	TGATGGATGATATGTGGCATT	21	5B_307	119	5B(-):398345117-398345235	-148.6
5B_329_248924647_248924667	TTCGCCGGATCAGCGTGCAGA	21	5B_329	52	5B(-):248924616-248924667	-157.6
5B_338_179241223_179241243	ATGGGTATATAGTGATCGTAT	21	5B_338	44	5B(-):179241223-179241266	-72.5
5B_357_91006944_91006964	TTTGAGACGAACACTGACCAA	21	5B_357	90	5B(-):91006944-91007033	-107.1
5B_41_66745825_66745845	AGGTCATGCTGGAGTTTCATC	21	5B_41	77	5B(+):66745769-66745845	-101.4
5B_63_248806644_248806664	TTCGCCGGAGCAGCGTGCAGA	21	5B_63	52	5B(+):248806644-248806695	-163.2
5B_70_295829251_295829272	TTTGGTTATTCATGTTGGAAAT	22	5B_70	127	5B(+):295829146-295829272	-62.9
5B_82_405104124_405104144	TTTGTCAAACTTGAAAGATTC	21	5B_82	62	5B(+):405104083-405104144	-67.9
5D_121_498588689_498588708	TGATGATATGATATTCGAGC	20	5D_121	105	5D(+):498588604-498588708	-66.6
5D_155_557400565_557400585	TAATCTCAACTCAACAGCCGC	21	5D_155	90	5D(+):557400496-557400585	-90.5
5D_157_557416042_557416062	TAATCTCAACTCAACAGCCGC	21	5D_157	90	5D(+):557415973-557416062	-84.1
5D_210_458932221_458932241	TTCGGACCAGGCTTCATTCCC	21	5D_210	74	5D(-):458932221-458932294	-64.3
5D_219_443310255_443310274	TGAGTAGGCCACGGGAGGCA	20	5D_219	72	5D(-):443310203-443310274	-95.8
5D_235_400242491_400242512	GGAGTGCCACTGAGAACACAAG	22	5D_235	82	5D(-):400242431-400242512	-117.2
5D_308_110367926_110367946	TCAAGAATCATATCATGGACA	21	5D_308	151	5D(-):110367926-110368076	-72.9
5D_311_83923113_83923133	TGGTCTGTGTTTGTTTCAAAC	21	5D_311	91	5D(-):83923043-83923133	-117
5D_315_63632614_63632634	AGGTCATGCTGGAGTTTCATC	21	5D_315	77	5D(-):63632614-63632690	-110.1
5D_321_29594848_29594868	CCTTCGCCGGCTGCGCGTTCC	21	5D_321	53	5D(-):29594816-29594868	-128.6
5D_343_5823675_5823695	TTGTTGGATGATAATATGCTA	21	5D_343	51	5D(-):5823645-5823695	-57.1

miRNA name	miRNA seq	miRNA len	pre-miRNA name	pre-miRNA len	pre position	energy
5D_67_275557432_275557452	CCTTCGCCGGCTGCGCGTTCC	21	5D_67	53	5D(+):275557432-275557484	-135.3
5D_88_397333362_397333382	ATTGCTGGATGATAATATGGT	21	5D_88	267	5D(+):397333362-397333628	-78.7
6A_101_560691899_560691919	GTCGGACCAGGCTTCATTCCC	21	6A_101	101	6A(+):560691819-560691919	-136.9
6A_117_601162219_601162239	AACAAGGATGAGGTTGATGTG	21	6A_117	64	6A(+):601162219-601162282	-68
6A_126_614539108_614539128	TTGATGGTTCAAGAAAGTCCT	21	6A_126	127	6A(-):614539108-614539234	-89.5
6A_179_437636987_437637008	GCTCACTGCTCTTCCTGTCATC	22	6A_179	240	6A(-):437636769-437637008	-117.6
6A_200_116961144_116961164	TGCCCTCGCCGGAGCAGCGTG	21	6A_200	61	6A(-):116961104-116961164	-176.7
6A_218_65549074_65549093	TGATGGGCAAGTCTGAGGTT	20	6A_218	83	6A(-):65549074-65549156	-62.1
6A_32_53784509_53784529	TCGCTTGGTGCAGATCGGGAC	21	6A_32	68	6A(+):53784509-53784576	-67.7
6A_38_67003286_67003306	TTGAATTTGATCATAGCATCA	21	6A_38	187	6A(+):67003120-67003306	-54.3
6A_71_349509324_349509344	ATCGTCCAGCAGTATCGTCTG	21	6A_71	122	6A(+):349509223-349509344	-101.4
6B_102_552498389_552498409	CCGCGGCGCCGTCTCCCGCTC	21	6B_102	122	6B(+):552498288-552498409	-100.8
6B_114_630041298_630041318	GTCGGACCAGGCTTCATTCCC	21	6B_114	119	6B(+):630041200-630041318	-139.8
6B_134_683241162_683241182	TGAGATGGTTTATGTCAAGCA	21	6B_134	227	6B(+):683241162-683241388	-90.7
6B_149_712109425_712109445	TTTTTGGATGTGCTCCTCTAG	21	6B_149	54	6B(+):712109392-712109445	-41.4
6B_168_715666018_715666038	TTGATGGTTCAAGAAAGTCCT	21	6B_168	78	6B(-):715665961-715666038	-69.5
6B_246_149480462_149480482	TCAATAGAAGCATGATTCAAC	21	6B_246	42	6B(-):149480462-149480503	-69.2
6B_257_103294205_103294225	TCGCTTGGTGCAGATCGGGAC	21	6B_257	68	6B(-):103294158-103294225	-67.7
6B_307_9848600_9848620	AAAAATACAGACGGTAAAGAA	21	6B_307	85	6B(-):9848536-9848620	-132.6

miRNA name	miRNA seq	miRNA len	pre-miRNA name	pre-miRNA len	pre position	energy
6B_31_46011293_46011313	TTTTGTTGGTTGTCATGTAAC	21	6B_31	45	6B(+):46011269-46011313	-62.7
6B_69_226138686_226138706	TGATAGATGACATGTGGCATT	21	6B_69	117	6B(+):226138686-226138802	-135.3
6B_74_290324168_290324188	TTCCCAAGATATGTTTCGAAT	21	6B_74	135	6B(+):290324054-290324188	-76.7
6B_94_448349806_448349827	GCTCACTGCTCTTCCTGTCATC	22	6B_94	89	6B(+):448349739-448349827	-134.4
6D_134_287888928_287888949	GCTCACTGCTCTTCCTGTCATC	22	6D_134	224	6D(+):287888928-287889151	-105.5
6D_142_366700175_366700195	CCGCGGCGCCGTCTCCCGCTC	21	6D_142	229	6D(+):366700175-366700403	-130.4
6D_155_418216733_418216753	GTCGGACCAGGCTTCATTCCC	21	6D_155	117	6D(+):418216637-418216753	-131.8
6D_184_465030232_465030252	TAGTTTTGGCATCAAATTCTT	21	6D_184	253	6D(+):465030000-465030252	-67.4
6D_186_469433478_469433498	TTGATGGTTCAAGAAAGTCCT	21	6D_186	258	6D(-):469433241-469433498	-75.3
6D_190_465169645_465169665	TGAAGTGGCAATTCTGGAAAA	21	6D_190	97	6D(-):465169645-465169741	-104.6
6D_203_454738883_454738903	AACAAGGATGAGGTTGATGTG	21	6D_203	49	6D(-):454738883-454738931	-56.9
6D_223_356801858_356801878	AAAAATGGCCCGGCCCGTAAA	21	6D_223	89	6D(-):356801790-356801878	-74.4
6D_93_43191745_43191765	TCGCTTGGTGCAGATCGGGAC	21	6D_93	68	6D(+):43191745-43191812	-67.4
7A_140_177170551_177170571	ATCAAGGATGAGGTTGATGTA	21	7A_140	65	7A(+):177170551-177170615	-54.4
7A_156_483486243_483486263	ATAGATGACATGTGGCATTCA	21	7A_156	114	7A(+):483486243-483486356	-107.4
7A_163_517872127_517872147	TTTTTGGCTGAGTTTTTTTTG	21	7A_163	271	7A(+):517871877-517872147	-61.4
7A_166_518772028_518772048	GTGCAGTTCTCCTCTGGCATG	21	7A_166	103	7A(+):518772028-518772130	-90
7A_174_594414043_594414063	TGATGGATGATATGTGGCATT	21	7A_174	119	7A(+):594414043-594414161	-146.7
7A_176_608412276_608412295	TGATAGATGATACGTGGCAT	20	7A_176	63	7A(+):608412276-608412338	-111.9

miRNA name	miRNA seq	miRNA len	pre-miRNA name	pre-miRNA len	pre position	energy
7A_179_615337209_615337229	TGAGAATATTCTTTATCTGTT	21	7A_179	265	7A(+):615336965-615337229	-87.5
7A_189_642148247_642148267	CCTTCGCCGGCTGCGCGTTCC	21	7A_189	51	7A(+):642148247-642148297	-115.6
7A_241_700479179_700479199	TGCCCTCGCCGGAGCAGCGTG	21	7A_241	61	7A(-):700479139-700479199	-186.3
7A_244_699802397_699802417	TGAGCGCACCGCCGTCGAGGG	21	7A_244	50	7A(-):699802397-699802446	-145.2
7A_261_677874162_677874182	TGCCCTCGCCGGAGCAGCGTG	21	7A_261	61	7A(-):677874122-677874182	-170.9
7A_283_647863765_647863785	TGATAGATGATATGTGGCATT	21	7A_283	117	7A(-):647863669-647863785	-140.5
7A_325_490282169_490282188	TGAGTAGGCCACGGGAGGCA	20	7A_325	72	7A(-):490282117-490282188	-114.7
7A_451_38967235_38967255	TTCGCCGGAGAAGCATGTTGC	21	7A_451	52	7A(-):38967204-38967255	-150.8
7A_54_62748055_62748076	TTTTGCTGGTTGAACGACCTCA	22	7A_54	118	7A(+):62748055-62748172	-124.5
7A_58_62755238_62755259	TTTTGCTGGTTGAACGACCTCA	22	7A_58	102	7A(+):62755238-62755339	-134.1
7B_224_655206237_655206257	TGAGATGGTTTATGTCAAGCA	21	7B_224	226	7B(-):655206032-655206257	-84.9
7B_227_653892319_653892339	AAGCATGATTCTGCTATTGAT	21	7B_227	134	7B(-):653892319-653892452	-121.2
7B_24_112632603_112632623	GCGAAGGATTTGCAGATACTC	21	7B_24	61	7B(+):112632603-112632663	-54
7B_257_501582237_501582257	GTGCAGTTCTCCTCTGGCATG	21	7B_257	91	7B(-):501582167-501582257	-88.3
7B_41_164100341_164100361	TTGGCATTGAGGGAGTCAAGC	21	7B_41	104	7B(+):164100341-164100444	-69
7B_58_258899118_258899138	TGAAGTGGTGATTCTGGAAAA	21	7B_58	99	7B(+):258899040-258899138	-118.6
7B_75_393128372_393128392	TGTCGACTGGAAGGACTCCAT	21	7B_75	270	7B(+):393128372-393128641	-83.9
7B_77_453147850_453147870	TATAAGACGTGTTGGACAGCT	21	7B_77	72	7B(+):453147850-453147921	-66.7
7B_85_550575456_550575475	TGAGTAGGCCACGGGAGACA	20	7B_85	72	7B(+):550575456-550575527	-99.1

miRNA name	miRNA seq	miRNA len	pre-miRNA name	pre-miRNA len	pre position	energy
7B_88_575377924_575377944	TGAGAATATTCTTTATCTGTT	21	7B_88	265	7B(+):575377680-575377944	-85.5
7B_98_620367736_620367756	ATGGATGATATGTGGCATTCA	21	7B_98	115	7B(+):620367736-620367850	-137.1
7D_118_150757026_150757046	GCGAAGGATTTGCAGATACTC	21	7D_118	61	7D(+):150757026-150757086	-54
7D_134_208677263_208677283	CCTTCGCCGGCTGCGCGTTCC	21	7D_134	53	7D(+):208677263-208677315	-134.2
7D_144_252246213_252246233	AAAATTTCCATGGCAGATGTA	21	7D_144	187	7D(+):252246213-252246399	-66.9
7D_146_266731158_266731178	TTGGCATCGAGGGAGTCAAGC	21	7D_146	204	7D(+):266731158-266731361	-74.6
7D_156_393579550_393579570	AGTTGAAGATGAGATATTGGA	21	7D_156	86	7D(+):393579550-393579635	-95.4
7D_183_535325364_535325384	TGAGAATATTCTTTATCTGTT	21	7D_183	264	7D(+):535325121-535325384	-84.3
7D_217_631615299_631615319	ATGAAGAGCGCGGGCAGCACA	21	7D_217	126	7D(+):631615299-631615424	-145
7D_223_631615399_631615419	ATGAAGAGCGCGGGCAGCACA	21	7D_223	126	7D(-):631615294-631615419	-127.4
7D_297_477101045_477101065	GTGCAGTTCTCCTCTGGCATG	21	7D_297	91	7D(-):477100975-477101065	-81.2
7D_301_467426692_467426712	CCTTCGCCGGCTGCGCGTTCC	21	7D_301	53	7D(-):467426660-467426712	-137.8
7D_307_447063867_447063887	TTAAAAATTGTGGATTTTTTT	21	7D_307	236	7D(-):447063652-447063887	-119.3
7D_310_421468223_421468243	TGAGCGCACCGCCGTCGAGGG	21	7D_310	50	7D(-):421468223-421468272	-146.1
7D_314_324277954_324277974	ATGTAGAAGCACCAAGGTAAG	21	7D_314	80	7D(-):324277954-324278033	-109.5
7D_316_312575192_312575212	TGAAGTGGCGATTTTGGAAAA	21	7D_316	95	7D(-):312575192-312575286	-113.3
7D_417_8968260_8968280	CCTTCGCCGGCTGCGCGTTCC	21	7D_417	53	7D(-):8968228-8968280	-155.5
7D_41_26984425_26984446	ATAAGCACCGATGCTTAGAGAA	22	7D_41	83	7D(+):26984364-26984446	-104
7D_49_34999043_34999062	TGAGTAGGCCACGGGAGGCA	20	7D_49	75	7D(+):34998988-34999062	-120.4

miRNA name	miRNA seq	miRNA len	pre-miRNA name	pre-miRNA len	pre position	energy
7D_5_1919120_1919140	ATGTGTATATAGTGATTGTAT	21	7D_5	44	7D(+):1919097-1919140	-74.9
7D_88_58259004_58259025	TTTTGCTGGTTGAACGACCTCA	22	7D_88	100	7D(+):58259004-58259103	-119.6
Un_168_465223151_465223171	TTACAGGGTGTAAACTCCACA	21	Un_168	220	Un(-):465223151-465223370	-57.8
Un_258_283159530_283159550	TCTCTGGCGGTAGGACTTACC	21	Un_258	69	Un(-):283159482-283159550	-120.8
Un_285_91214556_91214577	TTTTGCTGGTTGAACGACCTCA	22	Un_285	107	Un(-):91214471-91214577	-128.6
Un_293_77275220_77275240	ATGCACGATGATACTTCTAGC	21	Un_293	121	Un(-):77275120-77275240	-75.9
Un_70_242157156_242157176	CGTTTAGGCTGTTTGGACGTA	21	Un_70	52	Un(+):242157125-242157176	-114.2
Un_93_299838271_299838291	TGAAGTGGCAATTCTGGAAAA	21	Un_93	99	Un(+):299838193-299838291	-112
