# Supplementary material for: Differential Transcription Profiling Reveals the MicroRNAs Involved in Alleviating Damage to Photosynthesis under Drought Stress during the Grain Filling Stage in Wheat
Source: Int J Mol Sci. 2024 May 18;25(10):5518. doi: 10.3390/ijms25105518 (PMC11122533; doi:10.3390/ijms25105518)
Supplement: Supplementary file 1 [file ijms-25-05518-s001.zip › Supplementary Table S4.pdf]

Table S4 Drought-related miRNAs and their target genes

miRNA name	Significance of difference	target transcript id	target gene id	Significance of difference	target gene description
1A_255_13455352_13455372	Zhengmai1860 Significant decrease	TraesCS7A02G328600.1	TraesCS7A02G328600	Zhengmai1860 Significant increase	Pre-mRNA cleavage factor Im 25 kDa subunit 1
1A_255_13455352_13455372	Zhengmai1860 Significant decrease	TraesCS7D02G325500.1	TraesCS7D02G325500	Zhengmai1860 Significant increase	Pre-mRNA cleavage factor Im 25 kDa subunit 1
1D_104_371678191_371678211	Zhoumai18 Significant decrease	TraesCS3A02G315800.1	TraesCS3A02G315800	Zhoumai18 Significant increase	Diacylglycerol kinase
1D_104_371678191_371678211	Zhoumai18 Significant decrease	TraesCS3A02G315800.2	TraesCS3A02G315800	Zhoumai18 Significant increase	Diacylglycerol kinase
1D_104_371678191_371678211	Zhoumai18 Significant decrease	TraesCS3B02G351100.2	TraesCS3B02G351100	Zhoumai18 Significant increase	Diacylglycerol kinase
1D_104_371678191_371678211	Zhoumai18 Significant decrease	TraesCS3B02G351100.1	TraesCS3B02G351100	Zhoumai18 Significant increase	Diacylglycerol kinase
2D_165_612998307_612998326	Zhengmai1860 Significant increase	TraesCS7D02G515800.1	TraesCS7D02G515800	Zhengmai1860 Significant decrease	Probable magnesium transporter
3B_11_22107614_22107634	Zhoumai18 Significant increase	TraesCS3A02G020300.1	TraesCS3A02G020300	Zhoumai18 Significant decrease	Fructose-bisphosphate aldolase 3, chloroplastic
4B_79_658524855_658524875	Zhoumai18 Significant increase	TraesCS6A02G307700.1	TraesCS6A02G307700	Zhoumai18 Significant decrease	Geranylgeranyl diphosphate reductase, chloroplastic

miRNA name	Significance of difference	target transcript id	target gene id	Significance of difference	target gene description
4B_79_6585248 55_658524875	Zhoumai18 Significant increase	TraesCS6D0 2G286900.1	TraesCS6D 02G286900	Zhoumai18 Significant decrease	Geranylgeranyl diphosphate reductase, chloroplastic
6B_31_4601129 3_46011313	Zhengmai1860 Significant decrease	TraesCS4B0 2G116600.1	TraesCS4B 02G116600	Zhengmai1860 Significant decrease	HVA22-like protein
tae-miR159a	Zhoumai18 Significant increase	TraesCS1A0 2G217100.1	TraesCS1A 02G217100	Zhoumai18 Significant increase	PRA1 family protein
tae-miR159b	Zhoumai18 Significant increase	TraesCS1A0 2G217100.1	TraesCS1A 02G217100	Zhoumai18 Significant increase	PRA1 family protein
tae-miR531	Significant increase in Zhoumai 18 and Zhengmai 1860	TraesCS2B0 2G582300.1	TraesCS2B 02G582300	Significant decrease in Zhoumai 18 and Zhengmai 1860	A-type response regulator, Cytokinin signalin
tae-miR531	Significant increase in Zhoumai 18 and Zhengmai 1860	TraesCS2D0 2G552200.1	TraesCS2D 02G552200	Significant decrease in Zhoumai 18 and Zhengmai 1860	A-type response regulator, Cytokinin signalin
tae-miR531	Significant increase in Zhoumai 18 and Zhengmai 1860	TraesCS5D0 2G400700.1	TraesCS5D 02G400700	Zhengmai1860 Significant increase	Protein TORNADO 1
tae-miR9653b	Zhengmai1860 Significant increase	TraesCS7A0 2G461700.2	TraesCS7A 02G461700	Zhengmai1860 Significant decrease	Auxin response factor
tae-miR9653b	Zhengmai1860 Significant increase	TraesCS7A0 2G461700.1	TraesCS7A 02G461700	Zhengmai1860 Significant decrease	Auxin response factor

miRNA name	Significance of difference	target transcript id	target gene id	Significance of difference	target gene description
tae-miR9653b	Zhengmai1860 Significant increase	TraesCS7A02G461700.4	TraesCS7A02G461700	Zhengmai1860 Significant decrease	Auxin response factor
tae-miR9653b	Zhengmai1860 Significant increase	TraesCS7A02G461700.3	TraesCS7A02G461700	Zhengmai1860 Significant decrease	Auxin response factor
tae-miR9653b	Zhoumai18 Significant increase	TraesCS7D02G449900.2	TraesCS7D02G449900	Zhoumai18 Significant decrease	Auxin response factor
tae-miR9653b	Zhoumai18 Significant increase	TraesCS7D02G449900.3	TraesCS7D02G449900	Zhoumai18 Significant decrease	Auxin response factor
tae-miR9653b	Zhoumai18 Significant increase	TraesCS7D02G449900.1	TraesCS7D02G449900	Zhoumai18 Significant decrease	Auxin response factor
tae-miR9664-3p	Zhengmai1860 Significant increase	TraesCS5A02G201200.1	TraesCS5A02G201200	Zhengmai1860 Significant decrease	Zinc finger AN1 domain-containing stress-associated protein 12
tae-miR9664-3p	Zhengmai1860 Significant increase	TraesCS5B02G200000.1	TraesCS5B02G200000	Zhengmai1860 Significant decrease	Zinc finger AN1 domain-containing stress-associated protein 12
tae-miR9664-3p	Zhengmai1860 Significant increase	TraesCS5D02G207400.1	TraesCS5D02G207400	Zhengmai1860 Significant decrease	Zinc finger AN1 domain-containing stress-associated protein 12
4D_36_318329026_318329046	Zhoumai18 Significant decrease	TraesCS6D02G377900.1	TraesCS6D02G377900	Zhoumai18 Significant decrease	xanthophyll biosynthetic process

miRNA name	Significance of difference	target transcript id	target gene id	Significance of difference	target gene description
7A_179_615337209_615337229	Zhoumai18 Significant decrease	TraesCS6D02G377900.1	TraesCS6D02G377900	Zhoumai18 Significant decrease	xanthophyll biosynthetic process
7B_88_575377924_575377944	Zhoumai18 Significant decrease	TraesCS6D02G377900.1	TraesCS6D02G377900	Zhoumai18 Significant decrease	xanthophyll biosynthetic process
7D_183_535325364_535325384	Zhoumai18 Significant decrease	TraesCS6D02G377900.1	TraesCS6D02G377900	Zhoumai18 Significant decrease	xanthophyll biosynthetic process
